# Periscope: quantitative prediction of soluble protein expression in the periplasm of *Escherichia coli*

**DOI:** 10.1038/srep21844

**Published:** 2016-03-02

**Authors:** Catherine Ching Han Chang, Chen Li, Geoffrey I. Webb, BengTi Tey, Jiangning Song, Ramakrishnan Nagasundara Ramanan

**Affiliations:** 1 Chemical Engineering Discipline, School of Engineering, Monash University, Jalan Lagoon Selatan 46150, Bandar Sunway, Selangor, Malaysia; 2 Advanced Engineering Platform, School of Engineering, Monash University, Jalan Lagoon Selatan 46150, Bandar Sunway, Selangor, Malaysia; 3 Department of Biochemistry and Molecular Biology, Monash University, Melbourne VIC 3800, Australia; 4 Monash Centre for Data Science, Faculty of Information Technology, Monash University, Melbourne VIC 3800, Australia; 5 School of Chemistry, Monash University, Melbourne VIC 3800, Australia; 6 National Engineering Laboratory for Industrial Enzymes, Tianjin Institute of Industrial Biotechnology, Chinese Academy of Sciences, Tianjin 300308, China

## Abstract

Periplasmic expression of soluble proteins in *Escherichia coli* not only offers a much-simplified downstream purification process, but also enhances the probability of obtaining correctly folded and biologically active proteins. Different combinations of signal peptides and target proteins lead to different soluble protein expression levels, ranging from negligible to several grams per litre. Accurate algorithms for rational selection of promising candidates can serve as a powerful tool to complement with current trial-and-error approaches. Accordingly, proteomics studies can be conducted with greater efficiency and cost-effectiveness. Here, we developed a predictor with a two-stage architecture, to predict the real-valued expression level of target protein in the periplasm. The output of the first-stage support vector machine (SVM) classifier determines which second-stage support vector regression (SVR) classifier to be used. When tested on an independent test dataset, the predictor achieved an overall prediction accuracy of 78% and a Pearson’s correlation coefficient (PCC) of 0.77. We further illustrate the relative importance of various features with respect to different models. The results indicate that the occurrence of dipeptide glutamine and aspartic acid is the most important feature for the classification model. Finally, we provide access to the implemented predictor through the Periscope webserver, freely accessible at http://lightning.med.monash.edu/periscope/.

There has been a growing interest from researchers in expressing recombinant proteins in the periplasm of *E. coli*^1^,^2^,^3^,^4^,^5^,^6^,^7^,^8^, owing to the attractiveness of periplasmic expression. For example, periplasmic expression significantly facilitates downstream purification and produces target proteins with authentic N-terminal sequences upon proper cleavage of signal peptides^9^, which is otherwise not achievable through cytoplasmic expression. In addition, periplasm is the only oxidizing compartment in genetically unmodified *E. coli* cells and also the host to various chaperones and foldases^10^. These proteins and enzymes play crucial roles in facilitating proper protein folding and disulfide bond formation to ensure their biological functions.

The solubility of recombinant proteins upon expression in *E. coli* has been a main focus in protein expression studies. Overexpression of proteins generally gives high yield, but mostly in the form of inclusion bodies^11^,^12^. Despite the advantage of producing higher protein yield in the form of inclusion bodies, a series of onerous and expensive tasks are involved in resolubilizing the protein aggregates and refolding them^13^. Furthermore, there is no guarantee of retaining the biological activity of a protein after its refolding. Significant decrease in production yield upon refolding and purification is also common^9^. There are a number of examples of studies that report on the formation of inclusion bodies when proteins were heterologously expressed in the periplasm^14^,^15^,^16^,^17^, which suggest the possibility of inclusion body formation during periplasmic expression under certain conditions. On one hand, it is well-known that amino acid sequence is the major determinant of protein solubility^11^,^18^,^19^. The choice of signal peptide, on the other hand, has also been recognized as another important factor that strongly influences the protein secretion efficiency^20^ and also affects the protein expression yield in the periplasm. The rate of protein folding also influences the amount of proteins expressed, and can be estimated from the amino acid sequences^21^,^22^,^23^.

A number of computational algorithms and tools have been developed to predict both protein solubility and protein folding rate^21^,^23^,^24^,^25^,^26^ based on the correlations between amino acid sequence and these two important protein properties. For protein solubility prediction, classifiers are mainly built using SVM, while for real-valued protein folding rate prediction, multiple linear regressions or SVR are employed by most tools. Evaluation using cross-validation has revealed that a prediction accuracy of around 60–88% can be achieved for protein solubility prediction^27^, depending on the benchmark datasets. In general, the prediction tools for real-valued protein folding rate achieved correlation coefficients greater than 0.7^28^. More recently, a novel predictor that estimates the expression level and solubility of proteins in both *E. coli* and wheat germ cell-free expression systems has been established^29^. Among the three machine learning methods explored, SVM was chosen to train the prediction model. However, only qualitative prediction output is generated, rather than quantitative real-valued outputs, which are desirable and more practical for prioritizing the selection of candidates with better potentials from a pool of candidate proteins.

In the present work, we introduce a computational approach called Periscope (an acronym for **Peri**plasmic expre**s**sion **c**lassifier for s**o**luble **p**rotein **e**xpression) with a two-stage architecture, for quantitative prediction of soluble heterologous proteins in the periplasm of *E. coli*. More specifically, given the amino acid sequence of a signal peptide–target protein combination, Periscope is able to classify the soluble expression of the target protein into one of the three classes (high, medium, or low expression level) and further predict the quantity of soluble protein in the periplasm of *E. coli,* in the unit of mg/l.

## Results

### Architecture of the two-stage Periscope predictor

**Figure 1:**
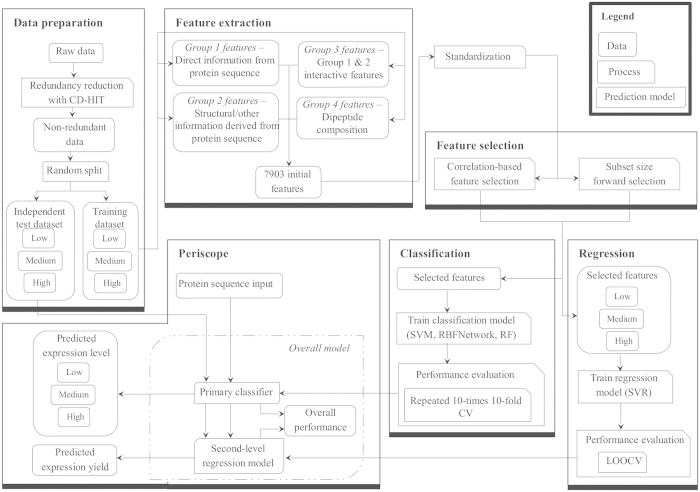
Overview of the Periscope development flowchart.

A flowchart that details the development of Periscope predictor is presented in Fig. 1. We designed a predictor with a two-stage architecture that first classifies an input sequence into high, medium or low expression level and subsequently estimates the soluble protein yield in the periplasm of *E. coli*. Researchers often report successful protein expression studies as high expression when hundreds mg/l of protein yield was achieved^30^,^31^. In addition, some published works also claimed protein yield between 10–50 mg/l as high level expression^32^,^33^,^34^,^35^. On the other hand, protein yield recorded in the range of 0.1–0.5 mg/l would be conventionally regarded as low expression level^32^. As such, in this study, the expression level of soluble proteins was categorized into three classes, namely, low (≤0.5 mg/l), medium (between 0.5 and 100 mg/l), and high (≥100 mg/l), in order to segregate the truly high expression level data from those moderately high expression level data. The first-stage predictor is based on a support vector classification (SVC) classifier that executes multi-class classification tasks, using the one-against-one approach. Previous work has identified the one-against-one approach as most practical^36^ compared with other approaches available for multi-class classification. Based on the output from the first-stage predictor, the second-stage predictor employs the SVR model of the assigned class to predict and output the real-valued expression yield. This two-stage predictor was specifically designed to address the issue of having expression yield data that varies up to 5-orders of magnitude. By building specific models for low, medium and high yield sequences, Periscope achieved real-valued expression yield prediction with greater accuracy.

### Performance of classification task

For the classification task, we benchmarked and compared the performance of SVM (implemented using LIBSVM package^37^) with two baseline algorithms-radial basis function network (RBFNetwork) and random forests (RF). The results are shown in Table 1. When assessed using repeated 10-times 10-fold cross validation (CV), the classification model built using LIBSVM achieved higher accuracy than the models built using RBFNetwork or RF. We used repeated *k-*fold CV to assess the accuracy of the classifier because it has been shown to reduce the bias, mean squared error and variance of *k*-fold CV^38^. The average accuracy, precision, F1-score and Matthew’s correlation coefficient (MCC) of the LIBSVM classification model topped both RBFNetwork and RF classification models by 6% and 14% on average, respectively. As a result, the LIBSVM classification model was employed as the primary classifier in the two-stage Periscope predictor.

### Performance of regression task

Three different regression models (low, medium and high regression models) were developed and employed in the second-layer predictor. Each model was trained using the training dataset that contained instances of respective class only. Owing to the relatively small sizes of the training datasets that were used to train these regression models, leave-one-out cross validation (LOOCV) was favored^38^,^39^ and used as the performance evaluation approach for various regression models. Table 2 summarizes the performance of respective models evaluated using LOOCV. The PCCs of these models ranged from 0.5–0.9. The mean absolute error (MAE) and root mean squared error (RMSE), on the other hand, ranged over 5-orders of magnitude. This was mainly attributed to the nature of the data where the real-valued soluble protein expression yield data ranged up to 5-orders of magnitude as well. In the second-stage predictor, the low regression model was developed to quantitatively predict the soluble protein expression yield of 0.5 mg/l and below. Conversely, the high regression model was trained to output real-valued soluble protein expression yield of 100 mg/l and above. Accordingly, both MAEs and RMSEs evaluated for low, medium and high regression models significantly differed from each other.

### Overall performance

In addition to the performance evaluation for individual first-stage classifiers and second-stage regression models, we also assessed the overall performance of Periscope. When tested on an independent test dataset with 15 unseen instances, the overall predictor recorded a prediction accuracy of 77.8% on average. Close correspondence was observed between the average accuracies of the overall predictor and the primary classifier built using LIBSVM. In general, the performance evaluated using independent tests offers greater trustworthiness as compared with various CV tests, by allowing strict assessment of the generalization capability of predictors on unseen data. This observation demonstrates the strong generalization capability of the developed overall predictor. Based on the correctly classified instances in the first-stage classifier, the second-stage regression model yielded PCC of 0.7726, MAE of 12.20, and RMSE of 20.38, respectively.

### Feature importance

From a total of 7,903 initial features, different feature subsets (Table 3) were selected for respective models, using correlation-based feature selection (CFS) approach, coupled with subset size forward selection as the search method0. CFS uses subsets of features and evaluates the corresponding significance by examining the predictive ability of individual feature and the redundancy between different features. Only subsets of features that are highly correlated with the class and at the same time poorly inter-correlated with one another were chosen. CFS, which is a *filter*-based approach, was used instead of the *wrapper*-based approach due to its suitability in handling small datasets compared with the *wrapper* method^40^. To further rank the relative importance of each feature in respective feature subsets, we evaluated the corresponding increase or decrease of each performance measure by removing a feature from the selected feature subset, one at a time, until each feature in the subset had been removed once (Tables 4 and 5). The occurrence of the dipeptide QD was shown to be the most important feature among the features selected for the primary classification task. When QD was removed from the feature subset, the MCC of the resultant classifier declined drastically (−81%) compared with the primary classifier trained using all seven features in the selected feature subset. Apart from MCC, other performance measures, such as precision, recall and F1-score of the resultant classifier also decreased by ~9–53%. Out of the seven features in the selected feature subset, the occurrence of the dipeptide CL least contributed to the improvement of the primary classifier’s performance. An insignificant performance improvement was observed in terms of accuracy, precision and F1-score, upon removing this feature from the selected feature subset. For the regression models in the second-stage predictor, the interaction between the T residue and maximum consecutive F residue, the dipeptide WQ, the interaction between the Y residue and the predicted protein folding rate were revealed as the most significant features for the high, medium and low regression models, respectively.

### Implementation of Periscope webserver

To provide access to the two-stage architecture predictor, an online webserver was developed and designed with a user-friendly interface (http://lightning.med.monash.edu/periscope/index.jsp). Tomcat7 handles the data preprocess and prediction for Periscope, deploying several JavaServer Pages (JSP) and Servlets. After a user submits amino acid sequence(s), Periscope performs the prediction using the constructed models and subsequently returns the predicted soluble protein expression level and yield in the periplasm of *E. coli*. It allows submission of up to five query sequences in FASTA format each submission. There is no limitation on the length of the query sequence. The usability of Periscope has been addressed in three important dimensions, namely, learnability, helpfulness and memorability. Examples of query sequences, particularly the format of single and multiple sequences, were illustrated in Periscope, to provide extended guidance to the users. Periscope is also equipped with detailed feedback when any error is detected during the submission of a query sequence. This is an important and useful function since error messages are a key part of communication between the web server and users. The source code of the predictor is downloadable from the web server (http://lightning.med.monash.edu/periscope/dataset.html), facilitating stand-alone application of Periscope and high-throughput prediction of large-scale sequence data. In addition to direct output retrieval displayed on the web page. Periscope offers an optional output delivery mode where users can retrieve the prediction output in a text file via email. This additional function allows the user to save the prediction output for interpretation or follow-up analysis.

## Discussion

During feature selection, the application of both CFS and subset size forward selection as the features selection approach resulted in a subset of seven features for the primary classification task (Table 3). Using similar approaches, different subsets of features were obtained for low, medium and high regression models in the second-stage predictor. Notably, features involving basic and positively charged residues (H, K, R) were repeatedly selected for the primary classification task. These include the occurrence frequency and maximum consecutive basic and positively charged residues. Despite the relatively high inter-correlation (r = 0.387) between these two features, the removal of either feature from the selected feature subset resulted in significant decrease in the classifier’s performance (Table 4). Previous research^29^ has also revealed similar significant effects of the basic property group residues on the solubility of the entire protein sequence and the C-terminal region of the protein sequence, which is in good agreement with the outcome of feature selection in this study. In addition, another group of researchers discovered a close correspondence between positively charged residues and protein solubility, in which they have highlighted the more prevalent influence of R residue as compared with K residue^41^. Importantly, the predicted protein folding rate was regarded as an important feature and selected in the feature subsets for the primary classifier and low regression model in the second-stage predictor, despite the possible noise introduced by inaccurate prediction. The important role of folding kinetics in determining the fate of the expressed protein as described in earlier studies^11^,^12^,^42^ is supported by the feature selection results in this study. A novel feature, dipeptide VT, has not been previously reported to show significant effect on either protein solubility or protein expression. The closest correspondence that has been reported was the negative influence of the repeating T and V residues on *E. coli* expression and solubility, respectively^29^. Based on the feature selection results in the current study, the dipeptide VT is the only feature that showed its relevance in both high and medium regression models in the second-stage predictor.

To further illustrate the competency of the developed predictor, we applied all 15 unseen instances in the independent test dataset to the Periscope predictor. Table 6 summarizes the prediction outcome of the independent test dataset. Out of 15 instances, 10 instances were correctly classified as medium expression level. The predicted yields for seven of these closely corresponded to the reported expression yield whereas the remaining predictions were observed to deviate in the range of 10–50 mg/l in expression yield. Due to misclassification of the stII–vtPA combination, Periscope predicted this combination to yield 9.254 mg/l of soluble vtPA. This combination was reported to produce 0.159 mg/l of soluble vtPA in the periplasm of *E. coli*^43^. Similar deviations in expression yield could be observed in other misclassified instances. Conversely, when instances were correctly classified, more accurate prediction of expression yield could be observed. When the exotoxin A from *Pseudomonas aeruginosa* was combined with ompA signal peptide, Periscope predicted this combination to yield 45 mg/l, realistically closer to the reported yield of 60 mg/l^36^. Another example is the elicitin 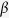-cinnamomin from *Phytophathora cinnamomi* that was merged with pelB signal peptide. This combination was reported to be expressed as soluble elicitin 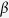-cinnamomin in the periplasm of *E. coli* at 13.3 mg/l^23^. Using pelB–elicitin 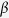-cinnamomin combination, Periscope predicted an expression yield of 7.3 mg/l. Apart from producing a few mediocre predictions, there were also some remarkably good predictions from Periscope. Firstly, the recombinant single-domain antibody fragment (V_H_ Hs) that specifically targets the cell receptor binding domain of the virulence factor produced by *Clostridium difficile*, named toxin B (TcdB), particularly B5.2, was reported to be expressed in soluble form at 6.7 mg/l, when fused to ompA signal peptide^44^. Periscope predicted the ompA-V_H_ Hs B5.2 combination to yield 6.8 mg/l of soluble protein in the periplasm of *E. coli*. The second example in this case study is the maltose-binding protein (MBP). When the native signal peptide of MBP was used along with the mature peptide, 9.8 mg/l of soluble MBP was obtained in the periplasm of *E. coli*^45^. Periscope predicted that 11.5 mg/l of soluble MBP will be produced in the periplasm of *E. coli* using the combined protein sequence of native MBP signal peptide and mature MBP. Evidently, Periscope correctly classified the expression levels of these two instances. Furthermore, the predicted soluble expression yield matched closely to the experimentally determined yield, with an average deviation of 0.9 mg/l. More examples from the independent test dataset were modified ompA–hPDI^46^ and ompA-V_H_Hs A19.2^27^ combinations, which also received outstandingly accurate predictions from Periscope. These results suggest that Periscope can be applied as a useful tool for prediction of soluble protein expression level and yield.

It has been more than a decade since researchers have started to venture into periplasmic expression for protein production. However, relatively fewer efforts have sought to improve the efficiency of periplasmic expression through rational selection of features. In the current work, we presented a computational approach to predict the expression level and yield of soluble protein in the periplasm of *E. coli*. With the aid of this predictor, rational selection of signal peptide–target protein combinations can be conducted with ease. Benchmarking experiments using repeated 10-times 10-fold CV and LOOCV indicate that Periscope is able to accurately classify the target sequence into one of the three classes (high, medium, or low expression level) and predict the amount of soluble protein in the periplasm of *E. coli.* However, user should be noted that Periscope is built based on a dataset that was curated through past literature. These literature data were reported based on various growth conditions and extraction protocols. In consideration of the average literature which were conducted based on shake flask fermentation and *E. coli* of type B strain, particularly BL21 (DE3) using strong promoter, these conditions were adopted as the reference conditions for all expression data in the dataset, as well as the predicted expression level and yield. Assumption was also made such that the three most commonly optimized parameters in a protein expression work, namely growth temperature, time of induction and concentration of inducer, had been optimized in respective literature data. The expression level and yield predicted by Periscope can be regarded as the soluble protein yield from the optimization of these three most common factors mentioned above.

One of the most challenging steps in building Periscope is the dataset generation process. Since the data were collected from various literatures, we faced difficulty in obtaining expression data that were reported under standardized conditions. In addition, majority of the literatures available have reported the expression data in the form of relative yield instead of absolute yield. These limitations prevented us from building a much larger dataset for improved training and testing of Periscope. Apart from the amino acid sequence-dependent factors that were considered in the current work, there are other biological and amino acid sequence-independent factors, such as optimization strategies in the omics level, that have been shown to affect the protein expression level. These biological and amino acid sequence-independent factors are highly accountable for the flaws and prediction discrepancies of Periscope. Regrettably, we were unable to incorporate these factors while building Periscope due to the absence of relevant information from literatures. As an effort to overcome these obstacles, we planned to extend our current work by building our own database with expression data generated under standardized conditions and at the same time allow the deposition of heterologous protein expression data in the form of absolute yield by other researchers. We are currently in the process of developing a vector database with different constructs to be offered to potential users for research purposes. Currently, Periscope is expected to be a powerful tool for quantitative prediction of protein expression level and rational selection of promising combinations of signal peptide–target proteins for the periplasmic expression of heterologous proteins in *E. coli*. In the future, with the ongoing plans and efforts, we hope to produce further release on our model, which will incorporate various biological and amino acid sequence-independent factors to serve for broader applications in proteomics.

## Methods

### Dataset generation

An exhaustive literature search was conducted *via* the National Center for Biotechnology Information (NCBI). Research papers annotated with descriptors including ‘soluble’, ‘recombinant protein’, ‘periplasm’ and ‘*Escherichia coli*’, were subjected to further scrutiny, aimed at identifying potential candidates that met the following prerequisites: (i) *E. coli* as the host, (ii) heterologous expression, (iii) containing signal peptide, (iv) expression targeted to the periplasm, (v) protein expression level quantitatively reported in terms of concentration (weight/volume) or equivalent, wherein the reported quantity can be converted into concentration, and (vi) accessible amino acid sequence information. The protein sequences of both signal peptide and target protein were determined and subjected to sequence redundancy reduction using the CD-HIT suite^47^ at 90% sequence identity. Sequence redundancy reduction is necessary to avoid potential overestimation of the performance and unreasonably high bias of the trained model^25^,^48^. In this work, high expression level was defined as soluble protein concentration of 100 mg/l or greater, whereas low expression level was defined as soluble protein concentration of 0.5 mg/l or less. Values amidst (between 0.5 and 100 mg/l) were categorized as medium expression level. Out of the 98 protein expression instances that remained after sequence redundancy reduction (Table S1), 16 were of high expression level (≤100 mg/l), 58 were classified as medium (between 0.5 and 100 mg/l) and 24 were classified as low (≤0.5 mg/l) expression levels, respectively. The dataset was randomly split into training and test datasets based on a ratio of 85%:15% between the training and test datasets. Both the training and test datasets were confirmed to include data from all three classes of the expression level.

### Feature extraction and feature selection

A total of 7,903 initial features were defined and extracted in this study. These were individual quantifiable properties that showed potential correlations with protein solubility, expression and folding rate^11^,^25^,^29^,^49^, based on existing knowledge or studies. These features could be divided into four major groups. Information that could be obtained directly from the amino acid sequence constituted the features in the first group. These include peptide length, occurrence frequencies of 20 amino acids, maximum count of consecutive identical amino acids, and occurrence frequencies of amino acids of the same physicochemical properties. The second group of features include structural and other features that were derived or calculated based on the amino acid sequence, including exposed residue fraction, contact number, propensities of alpha-helix (α-helix), beta-sheet (β-sheet) and coil. On the other hand, predicted features such as predicted protein folding rate, protein solubility, unfoldability and number of disordered residues were also used and belong to this feature group. The features included in the first and second groups added up to 122 features. The combinations between these features [(122 × 121)/2] contributed to 7381 non-redundant interactive features in the third group. The final category comprised of dipeptide composition features.

After determining all feature values for the 98 sequences in the dataset, these values were further standardized using the Z-score formula as shown below:


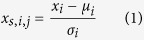


where *x*_*s,i,j*_ is the standardized value of the *i*-th feature for the *j*-th sequence, *x*_*i*_is the original value of the *i*-th feature for the *j*-th sequence, *μ*_*i*_ is the sample mean for the *i*-th feature and 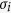 is the sample standard deviation for the *i*-th feature, respectively. The dataset was transformed to have zero mean and unit variance. This data processing step was employed in this work due to the nature of the dataset. The values of different features differ in a range up to 4-orders of magnitude. Additionally, these features had different units and scales. Accordingly, standardizing all data to achieve zero mean and unit variance was crucial to allow a fair comparison between them during feature selection by eliminating the dominating effect of features with greater numeric ranges over those with smaller numeric ranges. Features were selected using correlation-based feature selection^40^, coupled with subset size forward selection as the search method.

### Support vector machine for both classification and regression models

Both support vector classification (SVC) and support vector regression (SVR) algorithms in the LIBSVM package^37^ were employed to train the classification and regression models, respectively. Among the three types of kernels in LIBSVM, namely polynomial, radial basis function (RBF) and sigmoid, the RBF kernel was utilized to train the classification models. Optimization of the cost (*C*) and gamma (*γ*) parameters was conducted using an exhaustive grid search approach coupled with cross validation. Based on successful application of epsilon-SVR in previous studies^50^,^51^, epsilon-SVR was chosen to train the second-stage regression models in this work. RF and RBFNetwork were used as benchmarking algorithms in classification task. We used Weka software package^52^ for the implementation of the benchmarking algorithms. Same approach as described above was used to tune the hyperparameters of RF and RBFNetwork algorithms for generating optimal prediction performances.

### Performance Evaluation

The prediction performance of different models was assessed using both repeated 10-times 10-fold cross validation and leave-one-out cross validation, for classification models and regression models, respectively. In repeated 10-times 10-fold CV, the training data were randomly partitioned into 10 approximately equally sized subsets. At each cross-validation step, the model was trained with nine subsets while the remaining subset was used as the test dataset to evaluate the model’s performance. This procedure was repeated 10 times until each subset had been used as the test dataset once, to complete one cycle of the 10-fold CV. The randomization of training data was performed 10 times to conduct 10 cycles of 10-fold CV, with different combinations of data in each subset during each cycle. LOOCV, on the other hand, is an assessment approach where one single data was used to evaluate the performance of the model that was trained using the remaining data. This procedure was repeated *n* times until each sample in the dataset had been used as the test data once. For additional rigor, Periscope was further assessed in a case study using two examples that were withheld in the independent test dataset.

Different performance measures were employed in this work in order to comprehensively assess the performance of the developed model. This is because none of the single performance measure alone can well describe all aspects of a predictor’s performance^53^. Different from binary classification, the primary classification model developed in this study is a multi-class classifier (e.g. high, medium and low expression level). In cases where there are more than two classes, typical mathematical formulas for performance measures such as accuracy, error rate, precision, recall, F1-score and MCC are no longer applicable^53^. To address this, the generalized mathematical formulas of respective performance measures were adopted where either macro-averaging or micro-averaging was conducted. The former approach determines the average measure by summing up individual measures for each class, whereas the latter approach computes the average measure using cumulative true positive (*TP*), true negative (*TN*), false positive (*FP*) and false negative (*FN*) of the overall model. *TP* is the number of correctly predicted positive instances. Similarly, *TN, FP* and *FN* are the number of correctly predicted negative instances, number of instances that are incorrectly predicted as positive instances, and number of instances that are incorrectly predicted as negative instances, respectively. In this work, macro-averaging was employed because this approach treats all classes equally compared with micro-averaging that favors larger classes^54^.

The generalized mathematical formulas of different performance measures were provided below:


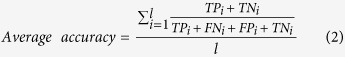



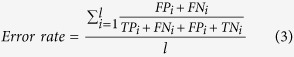



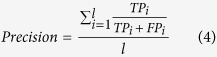



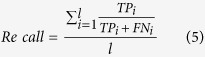



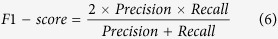



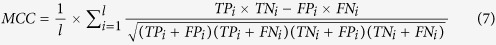


where *i* is the index representing the low, medium and high expression level classes, while *l* is the total number of classes available. MCC indicates the correlation coefficient between the assigned and actual classes of respective instances in the dataset.

For the real-valued regression task, the prediction performance was evaluated using PCC, MAE, and RMSE, as shown below:


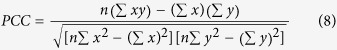



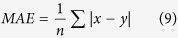



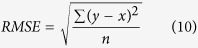


where *x* is the actual soluble protein expression value, *y* is the predicted soluble protein expression value, while *n* is the number of instances subjected to the prediction. PCC determines the correlation between the predicted and actual values. MAE and RMSE, on the other hand, describe the extent of deviation of the predicted values, with reference to the actual experimentally reported values in the dataset.

## Additional Information

**How to cite this article**: Chang, C. C. H. *et al*. Periscope: quantitative prediction of soluble protein expression in the periplasm of *Escherichia coli. Sci. Rep.* **6**, 21844; doi: 10.1038/srep21844 (2016).

## Supplementary Material

Supplementary Information

## Figures and Tables

**Table 1:** Performance comparison of primary classifiers developed using different machine learning algorithms.

Algorithm	LIBSVM	RBFNetwork	RF
Average accuracy	**0.7904**	0.7647	0.7727
Error rate	**0.2096**	0.2353	0.2273
Precision	**0.7272**	0.6098	0.6085
Recall	0.6218	**0.6509**	0.5390
F1 score	**0.6704**	0.6297	0.5717
MCC	**0.4760**	0.4371	0.3623

Performance was evaluated based on repeated 10-times 10-fold CV. The highest score within the same performance measure category is indicated in bold font with the exception of error rate where the lowest score is indicated in bold font.

**Table 2:** Performance of second-stage regression models.

Regression model	PCC	MAE	RMSE
Low	0.6934	0.0728	0.0845
Medium	0.5386	9.81	16.91
High	0.9381	425.81	593.54

**Table 3:** Feature subsets selected for both classification and regression tasks and the description of respective features.

Task	Selected features	Description
CLASSIFICATION	BPC	Occurrence frequency of **b**asic and **p**ositively **c**harged residue (H, K, R)
	Sulfur	Occurrence frequency of **sulfur**-containing residue (C, M)
	MCBPC	**M**aximum **c**onsecutive **b**asic and **p**ositively **c**harged residue (H, K, R)
	logPFR	**P**rotein **f**olding **r**ate in **log**10 base, predicted using SeqRate
	CL	Occurrence of dipeptide cysteine and leucine
	QD	Occurrence of dipeptide glutamine and aspartic acid
	VE	Occurrence of dipeptide valine and glutamic acid
REGRESSION HIGH	TP	Occurrence of dipeptide threonine and proline
	VT	Occurrence of dipeptide valine and threonine
	T × MCPhe	Occurrence frequency of threonine interacting with **m**aximum **c**onsecutive **phe**nylalanine residue
REGRESSION MEDIUM	ER	Occurrence of dipeptide glutamic acid and arginine
	WQ	Occurrence of dipeptide tryptophan and glutamine
	VT	Occurrence of dipeptide valine and threonine
	R × AbsCharge	Occurrence frequency of arginine interacting with **abs**olute **charge** per residue
	ANC × MCAliphatic	Occurrence frequency of **a**cidic and **n**egatively **c**harged residues interacting with **m**aximum **c**onsecutive **aliphatic** residue (I, L, V, A, G)
	MCCys × pI	**M**aximum **c**onsecutive **cys**teine residue interacting with isoelectric point (pI)
REGRESSION LOW	F × logPFR	Occurrence frequency of phenylalanine interacting with **p**rotein **f**olding **r**ate in **log**10 base, predicted using SeqRate^55^
	S × MCNPH	Occurrence frequency of serine interacting with **m**aximum **c**onsecutive **n**on-**p**olar and **h**ydrophilic residue (I, L, V, A, G, P)
	y × transmembrane	Occurrence of tyrosine interacting with occurrence of transmembrane, predicted using TMHMM^56^
	Y × nlogPFR	Occurrence frequency of tyrosine interacting with **p**rotein **f**olding **r**ate in **n**atural **log** base, predicted using SeqRate

**Table 4:** Relative significance of features selected (descending order) for primary classification task.

Feature removed	Percentage change				
	Accuracy	Precision	Recall	F1score	MCC
QD	−9.25	−49.94	−52.85	−43.20	−81.10
BPC	−5.28	−25.94	−37.22	−20.52	−47.77
Sulfur	−3.66	−10.74	−30.53	−8.62	−31.09
VE	−3.35	−6.15	−29.40	−5.76	−26.70
MCBPC	−2.13	−12.07	−30.18	−8.97	−26.28
logPFR	−1.12	−2.26	−24.46	−0.33	−14.81
CL	1.02	5.38	−19.80	6.52	−2.85

Percentage changes were evaluated using repeated 10 times 10-fold CV, with reference to the classification model trained using all seven features selected.

**Table 5:** Relative significance of features selected (descending order) for second-level regression models.

Regression Models	Feature removed	Percentage change		
		PCC	MAE	RMSE
High	T × MCPhe	−3.96	39.83	36.49
	TP	−2.32	24.85	14.90
	VT	−1.98	22.43	26.52
Medium	WQ	−52.35	55.83	40.49
	ANC × MCAliphatic	−47.28	35.51	27.50
	ER	−35.52	26.77	16.13
	R × AbsCharge	−15.74	6.28	7.41
	VT	−2.04	13.28	3.71
	MCCys × pI	−4.90	6.88	3.49
Low	Y × nlogPFR	−36.06	15.54	17.17
	S × MCNPH	−2.54	11.31	5.08
	y × transmembrane	6.02	5.60	0.76
	F × logPFR	2.90	0.36	−2.14

Percentage changes were evaluated using LOOCV, with reference to the high, medium and low regression models trained using respective feature subsets.

**Table 6:** Experimental and predicted expression data of independent test dataset.

Protein	Signal peptide	Experimental results		Predicted results from Periscope		
		Expression level	Yield (mg/l)	Expression level	Expression level classification matrix [High,Low,Medium]	Yield (mg/l)
V_H_Hs B5.2	ompA	Medium	6.7^44^	Medium	0.09,0.15,0.75	6.8009
scFv13.R4	TorA	Low	0.06^57^	Medium	0.09,0.27,0.64	4.6039
Human protein disulfide isomerase (hPDI)	modified from ompA	Medium	30^46^	Medium	0.10,0.18,0.72	29.8987
Granulocyte-macrophage colony-stimulating factor (GM-CSF)	CSP	High	800^58^	Medium	0.06,0.33,0.61	14.7816
V_H_Hs A5.1	ompA	Medium	55.5^44^	Medium	0.05,0.20,0.75	5.3871
Maltose-binding protein (MBP)	native	Medium	9.8^12^	Medium	0.39,0.12,0.49	11.6017
human epidermal growth factor (hEGF)	phoA	Medium	1.026^59^	Medium	0.25,0.35,0.40	11.2143
enzymatically active version of tissue plasminogen activator (vtPA)	stII	Low	0.159^43^	Medium	0.08,0.44,0.48	9.2542
Cellulose binding domain (CBD)	Cex	High	5310^60^	Medium	0.16,0.30,0.54	2.013
Exotoxin A from *Pseudomonas aeruginosa*	ompA	Medium	60^61^	Medium	0.39,0.21,0.40	45.1823
V_H_Hs A19.2	ompA	Medium	3.8^44^	Medium	0.06,0.13,0.81	4.0973
Single-chain antibody Fv fragment (scFv)	mBiP	High	115^62^	Medium	0.22,0.38,0.41	12.0768
V_H_Hs B7.3	ompA	Medium	1.5^27^	Medium	0.06,0.31,0.63	4.4261
Glutaminase from *Bacillus licheniformis* DSM13	ompA	Medium	80^63^	Medium	0.10,0.25,0.65	44.384
elicitin beta-cinnamomin from *Phytophathora cinnamomi*	pelB	Medium	13.3^64^	Medium	0.09,0.29,0.62	7.2501
