# Supplementary material for: Periscope: quantitative prediction of soluble protein expression in the periplasm of Escherichia coli
Source: Sci Rep. 2016 Mar 2;6:21844. doi: 10.1038/srep21844 (PMC4773868; doi:10.1038/srep21844)
Supplement: Supplementary Information [file srep21844-s1.pdf]

Periscope: quantitative prediction of soluble protein expression in the periplasm of *Escherichia coli*

Supplementary Information

Catherine Ching Han Chang[1,3], Chen Li[3], Geoffrey I. Webb[4], BengTi Tey[1,2], Jiangning Song[3,4,6*] and Ramakrishnan Nagasundara Ramanan[1,2,5*]

[1]Chemical Engineering Discipline, [2]Advanced Engineering Platform, School of Engineering, Monash University, Jalan Lagoon Selatan, 46150 Bandar Sunway, Selangor, Malaysia. [3]Department of Biochemistry and Molecular Biology, [4]Monash Centre for Data Science, Faculty of Information Technology, [5]School of Chemistry, Monash University, Melbourne, VIC 3800, Australia, [6]National Engineering Laboratory for Industrial Enzymes, Tianjin Institute of Industrial Biotechnology, Chinese Academy of Sciences, Tianjin 300308, China

No.	Protein	Signal peptide	Signal peptide - target protein sequences	Expression yield	Ref.
1	xylanase, *xynA2*	ompA	MKKTAIAIAVALAGFATVAQAMQTTPNSEGWHDGYYYSWWSDGGAQ ATYTNLEGGTYEISWGDGGNLVGGKGWNPGLNARAIHFEGVYQPNG NSYLAVYGWTRNPLVEYYIVENFGTYDPSSGATDLGTVECDGSIYRLG KTTRVNAPSIDGTQTFDQYWSVRQDKRTSGTVQTGCHFDAWARAGL NVNGDHYYQIVATEGYFSSGYARITVADVG	692.8 mg/l	1
2	human interleukin 6	phoA	MKQSTIALALLPLLFTPVTKAMPVPPGEDSKDVAAPHRQPLTSSERIDK QIRYILDGISALRKETCNKSNMCESSKEALAENNLNLPKMAEKDGCFQ SGFNEETCLVKIITGLLEFEVYLEYLQNRFESSEEQARAVQMSTKVLIQ FLQKKAKNLDAITTPDPTTNASLLTKLQAQNQWLQDMTTHLILRSFKEF LQSSLRALRQMHHHHHH	ND	2

3	*Candida Antarctica* lipase B (CALB)	pelB	MKYLLPTAAAGLLLLAAQPAMALPSGSDPAFSQPKSVLDAGLTCQGASPSSVSKPILLVPGTGTTGPQSFDSNWIPLSTQLGYTPCWISPPPFMLNDTQVNTEYMVNAITALYAGSGNNKLPVLTWSQGGLVAQWGLTFFPSIRSKVDRLMAFAPDYKGTVLAGPLDALAVSAPSVWQQTTGSALTTALRNAGGLTQIVPTTNLYSATDEIVQPQVSNSPLDSSYLFNGKNVQAQAVCGPLFVIDHAGSLTSQFSYVVGRSALRSTTGQARSADYGITDCNPLPANDLTPEQKVAAAALLAPAAAAIVAGPKQNCEPDLMPYARPFAVGKRTCSGIVTP	5.2 mg/l culture	[3]
4	human prolactin (hPRL)	DsbA	MKKIWLALAGLVLAFSASALPICPGGAARCQVTLRDLFDRAVVLSHYIHNLSSEMFSEFDKRYTHGRGFITKAINSCHTSSLATPEDKEQAQQMNQKDFLSLIVSILRSWNEPLYHLVTEVRGMQEAPEAILSKAVEIEEQTKRLLEGMELIVSQVHPETKENEIYPVWSGLPSLQMADEESRLSAYYNLLHCLRRDSHKIDNYLKLLKCRIIHNNNC	2.8 mg/l	[4]
5	Epstein-Barr virus (EBV) interleukin-10 (EBV IL-10)	ompF	MKRNILAVIVPALLVAGTANAFPQMLRDLRDAFSRVKTFFQTKDEVDNLLLKESLLEDFKGYLGCQALSEMIQFYLEEVMPQAENQDPEAKDHVNSLGENLKTLRLRLRRCHRFLPCENKSKAVEQIKNAFNKLQEKGIYKAMSEFDIFINYIEAYMTIKAR	0.0678 mg/l	[5]
6	Human Cytomegalovirus (HCMV) interleukin-10 (HCMV IL-10)	ompF	MKRNILAVIVPALLVAGTANASEEAKPATTTTIKNTKPQCRPEDYATRLQDLRVTFHRVKPTLQREDDYSVWLDGTVVKGCWGCSVMDWLLRRYLEIVFPAGDHVYPGLKTELHSMRSTLESIYKDMRQCPLLGCGDKSVISRLSQEAERKSDNGTRKGLSELDTLFSRLEEYLHSRK	1.5 mg/l	[5]

7	Potein A - acidic mammalian chitinase (AMCase)-V5-His	truncated form of Protein A	MKKKNIYSIRKLGVGIASVTLGTLLISGGVTPAANAAQHDEAVDNKFNKEQQNAFYEILHLPNLNEEQRNAFIQSLKDDPSQSANLLAEAKKLNDAQAPKVDNKFNKEQQNAFYEILHLPNLNEEQRNAFIQSLKDDPSQSANLLAEAKKLNDAQAPKVDANSYNLICYFTNWAQYRPGLGSFKPDDINPCLCTHLIYAFAGMQNNEITTIEWNDVTLYKAFNDLKNRNSKLKTLLAIGGWNFGTAPFTTMVSTSQNRQTFITSVIKFLRQYGFDGLDLDWEYPGSRGSPPQDKHLFTVLVKEMREAFEQEAIESNRPRLMVTAAVAGGISNIQAGYEIPELSKYLDFIHVMTYDLHGSWEGYTGENSPLYKYPTETGSNAYLNVDYVMNYWKNNGAPAEKLIVGFPEYGHTFILRNPSDNGIGAPTSGDGPAGPYTRQAGFWAYYEICTFLRSGATEVWDASQEVPYAYKANEWLGYDNIKSFSVKAQWLKQNNFGGAMIWAIDLDDFTGSFCDQGKFPLTSTLNKALGISTEGCTAPDVPSEPVTTPPGSGSGGGSSGGSSGGSGFCADKADGLYPVADDRNAFWQCINGITYQQHCQAGLVFDTSCNCCNWPARGHPFEGKPIPNPLLGLDSTRTGHHHHHH	1.527 mg/l	[6]
8	Chitinase precursor of 45 kDa from *C. violaceum* (*Cv*Chi45)	native	MRRTTGRAIAMAMLLALGQHAWAAACPGWAEGTAYKVGDVVSYNNANYTALVAHTAYVGANWNPAASPTLWTPGGSCAGGDPTPPTPPNPPTPPSPPPGNTVPFAKHALVGYWHNFANPSGSAFPLSQVSADWDVIVVAFADDAGNGNVSFTLDPAAGSAAQFIQDIRAQQAKGKKVVLSLGGQNGSVTLNNATQVQNFVNSLYGILTQYGFDGIDLDLESGSGIVVGAPVVSNLVSAVKQLKAKIGPNFYLSMAPEHPYVQGGFVAYGGNWGAYLPIIDGLRDDLSVIHVQYYNNGGLYTPYSTGVLAEGSADMLVGGSKMLIEGFPIANGASGSFKGLRPDQVAFGVPSGRSSANSGFVTADTVAKALTCLTTLQGCGSVKPAQAYPAFRGVMTWSINWDRRDGYTFSRPVAASLRQQPVAAQAGKKKAARATRTAWHHHHHH	ND	[7]

9	fusion protein human alpha-synuclein + transduction domain of Tat protein from HIV (TAT-AS)	Tat protein form HIV	MMRGSHHHHHHGMARGYGRKKRRPASPGASMMHHHHHHMDVFMK<br>GLSKAKEGVVAAAEKTKQGVAEAAGKTKEGVLYVGSKTKEGVVHGVA<br>TVAEKTKEQVTNVGGAVVTGVTAVAQKTVEGAGSIAAATGFVKKDQL<br>GKNEEGAPQEGILEDMPVDPDNEAYEMPSEEGYQDYEPEA	20 mg/l	[8]
10	Fusion protein sSpAD-Gfpmut3.1	sSpAD	ADAQQNKFNKDQQSAFYEILNMPNLNEEQRNGFIQSLKDDPSQSTNV<br>LGEAKKLNESQAPK<br>MSKGEELFTGVVPILVELDGDVNGHKFSVSGEGEGDATYGKLTLKFIC<br>TTGKLPVPWPTLVTTFSYGVQCFSRYPDHMKQHDFFKSAMPEGYVQE<br>RTIFFKDDGNYKTRAEVKFEGDTLVNRIELKGIDFKEDGNILGHKLEYNY<br>NSHNVYIMADKQKNGIKVNFKIRHNIEDGSVQLADHYQQNTPIGDGPVL<br>LPDNHYLSTQSALSKDPNEKRDHMVLLEFVTAAGITHGMDELYK	1.3 mg/l	[9]
11	Fusion protein sSpAD-SOD (Human superoxide dismutase)	sSpAD	ADAQQNKFNKDQQSAFYEILNMPNLNEEQRNGFIQSLKDDPSQSTNV<br>LGEAKKLNESQAPKMATKAVCVLKGDGPVQGIINFEQKESNGPVKVW<br>GSIKGLTEGLHGFHVHEFGDNTAGCTSAGPHFNPLSRKHGGPKDEER<br>HVGDLGNVTADKDGVADVSIEDSVISLSGDHCIIGRTLVVHEKADDLGK<br>GGNEESTKTGNAGSRLACGVIGIAQL	16.4 mg/l	[9]

12	19-kDa antigen of *Mycobacterium bovis* AN5	first 36aa of MPB70	MKVKNTIAATSFAAAGLAALAVAVSPPAAAGDLVGPTSSNKSTTGSGETTTAAGTTASPGAASGPKVVIDGKDQNVTGSVVCTTAAGNVNIAIGGAATGIAAVLTDGNPPEVKSVGLGNVNGVTLGYTSGTGQGNASATKDGSHYKITGTATGVDMANPMSPVNKSFEIEVTCS	2.5 mg/l	[10]
13	19-kDa antigen of *Mycobacterium bovis* AN5	first 40 aa of alpha-peptide of beta-galactosidase	MTMITPNSSSVPGDPLESTCRHASLLAVVLQRRDWENPGVGDLVGPTSSNKSTTGSGETTTAAGTTASPGAASGPKVVIDGKDQNVTGSVVCTTAAGNVNIAIGGAATGIAAVLTDGNPPEVKSVGLGNVNGVTLGYTSGTGQGNASATKDGSHYKITGTATGVDMANPMSPVNKSFEIEVTCS	ND	[10]
14	Recombinant hydrolase from *Thermobifida fusca* (rTfH)	ompA	MKKTAIAIAVALAGFATVAQAMANPYERGPNPTDALLEASSGPFSVSEENVSRLSASGFGGGTIYYPRENNTYGAVAISPGYTGTEASIAWLGERIASHGFVVITIDTITTLDQPDSRAEQLNAALNHMINRASSTVRSRIDSSRLAVMGHSMGGGGTLRKASQRPDLKAAIPLTPWHLNKNWSSVTVPTLIIGADLDTIAPVATHAKPFYNSLPSSISKAYLELDGATHFAPNIPNKIIGKYSVAWLKRFVDNDTRYTQFLCPGPRDGLFGEVEEYRSTCPF	587 mg/l	[11]
15	MBP	native	MKIKTGARILALSALTTMMFSASALAKIEEGKLVIWINGDKGYNGLAEVGKKFEKDTGIKVTVEHPDKLEEKFPQVAATGDGPDIIFWAHDRFGGYAQSGLLAEITPDKAFQDKLYPFTWDAVRYNGKLIAYPIAVEALSLIYNKDLLPNPPKTWEEIPALDKELKAKGKSALMFNLQEPYFTWPLIAADGGYAFKYENGKYDIKDVGVDNAGAKAGLTFLVDLIKNKHMNADTDYSIAEAAFNKGETAMTINGPWAWSNIDTSKVNYGVTVLPTFKGQPSKPFVGVLSAGINAASPNKELAKEFLENYLLTDEGLEAVNKDKPLGAVALKSYEEELAKDPRIAATMENAQKGEIMPNIPQMSAFWYAVRTAVINAASGRQTVDEALKDAQTRITK	9.8 mg/l	[12]

16	MBP-GFP	TorA	MNNNDLFQASTTTFLAQLGGLTVAGMLGPSLLTPRRATAKIEEGKLVI WINGDKGYNGLAEVGKKFEKDTGIKVTVEHPDKLEEKFPQVAATGDG PDIIFWAHDRFGGYAQSGLLAEITPDKAFQDKLYPFTWDAVRYNGKLIA YPIAVEALSLIYNKDLLPNPPKTWEEIPALDKELKAKGKSALMFNLQEPY FTWPLIAADGGYAFKYENGKYDIKDVGVDNAGAKAGLTFLVDLIKNKH MNADTDYSIAEAAFNKGETAMTINGPWAWSNIDTSKVNYGVTVLPTFK GQPSKPFVGVLSAGINAASPNKELAKEFLENYLLTDEGLEAVNKDKPL GAVALKSYEEELAKDPRIAATMENAQKGEIMPNIPQMSAFWYAVRTAVI NAASGRQTVDEALKDAQTRITKRKGEELFTGVVPILVELDGDVNGHKF SVSGEGEGDATYGKLTLKFICTTGKLPVPWPTLVTTFGYGVQCFARYP DHMKQHDFFKSAMPEGYVQERTIFFKDDGNYKTRAEVKFEGDTLVNRI ELKGIDFKEDGNILGHKLEYNYNSHNVYIMADKQKNGIKVNFKIRHNIED GSVQLADHYQQNTPIGDGPVLLPDNHYLSTQSALSKDPNEKRDHMVL LEFVTAAGITHGMDELYK	2.6 mg/l	12
17	scFv13.R4	TorA	MNNNDLFQASTTTFLAQLGGLTVAGMLGPSLLTPRRATAMAEVQLVE SGGSLVKPGGSLRLSCAASGFTFSNYSMNWVRQAPGKGLEWISSISG SSRYIYYADFVKGRFTISRDNATNSLYLQMNSLRAEDTAVYYCVRSSITI FGGGMDVWGRGTLVTVSSGGGGSGGGGSGGGGSQSVLTQPASVS GSPGQSITISCAGTSSDVGGYNYVSWYQQHPGKAPKLMIYEDSKRPS GVSNRFSGSKSGNTASLTISGLQAEDEADYYCSSYTTRSTRVFGGGT KLAVLGAAAEQKLISEEDLNGAAHHHHHH	0.06 mg/l	12
18	MBP-scFv13.R4	native MBP	MKIKTGARILALSALTTMMFSASALAKIEEGKLVIWINGDKGYNGLAEVG KKFEKDTGIKVTVEHPDKLEEKFPQVAATGDGPDIIFWAHDRFGGYAQ SGLLAEITPDKAFQDKLYPFTWDAVRYNGKLIAYPIAVEALSLIYNKDLL PNPPKTWEEIPALDKELKAKGKSALMFNLQEPYFTWPLIAADGGYAFK YENGKYDIKDVGVDNAGAKAGLTFLVDLIKNKHMNADTDYSIAEAAFNK GETAMTINGPWAWSNIDTSKVNYGVTVLPTFKGQPSKPFVGVLSAGIN AASPNKELAKEFLENYLLTDEGLEAVNKDKPLGAVALKSYEEELAKDP RIAATMENAQKGEIMPNIPQMSAFWYAVRTAVINAASGRQTVDEALKD AQTRITKMAEVQLVESGGSLVKPGGSLRLSCAASGFTFSNYSMNWVR QAPGKGLEWISSISGSSRYIYYADFVKGRFTISRDNATNSLYLQMNSLR AEDTAVYYCVRSSITIFGGGMDVWGRGTLVTVSSGGGGSGGGGSGG GGSQSVLTQPASVSGSPGQSITISCAGTSSDVGGYNYVSWYQQHPGK APKLMIYEDSKRPSGVSNRFSGSKSGNTASLTISGLQAEDEADYYCSS YTTRSTRVFGGGTKLAVLGAAAEQKLISEEDLNGAAHHHHHH	8.4 mg/l	12

19	Hemagglutinin (HA) receptor binding domain	Native ecotin	MKTILPAVLFAAFATTSAWAHHHHHHDDDDKGVAPLHLGKCNIAGWIL GNPECESLSTASSWSYIVETSSSDNGTCYPGDFIDYEELREQLSSVSS FERFEIFPKTSSWPNHDSNKGVTAACPHAGAKSFYKNLIWLVKKGNSY PKLSKSYINDKGKEVLVLWGIHHPSTSADQQSLYQNADAYVFVGSSRY SKKFKPEIAIRPKVRDQEGRMNYYWTLVEPGDKITFEATGNLVVPRYA FAMERNAGSGIIISD	10 mg/l	[13]
20	Disulfide-rich sea anemone peptide (APETx2) from *Anthopleura elegantissima*	MalE	MKIKTGARILALSALTTMMFSASALAHHHHHHKIEEGKLVIWINGDKGY NGLAEVGKKFEKDTGIKVTVEHPDKLEEKFPQVAATGDGPDIIFWAHD RFGGYAQSGLLAEITPDKAFQDKLYPFTWDAVRYNGKLIAYPIAVEALS LIYNKDLLPNPPKTWEEIPALDKELKAKGKSALMFNLQEPYFTWPLIAA DGGYAFKYENGKYDIKDVGVDNAGAKAGLTFLVDLIKNKHMNADTDYS IAEAAFNKGETAMTINGPWAWSNIDTSKVNYGVTVLPTFKGQPSKPFV GVLSAGINAASPNKELAKEFLENYLLTDEGLEAVNKDKPLGAVALKSYE EELAKDPRIAATMENAQKGEIMPNIPQMSAFWYAVRTAVINAASGRQT VDEALKDAQTRITKEKLYFQGAGTACSCGNSKGIYWFYRPSCPTDRG YTGSCRYFLGTCCTPAD	1 mg/l	[14]
21	Variable light chain single domain antibody (V_L $dAb_{delta115}$) truncated after light chain residue 115	ompA	MKKTAIAIAVALAGFATVAQADIQMTQSPSSLSASVGDRVTITCRASQDI SNYLSWYQQKPGKAPKLLIYYTSKLHSGVPSRFSGSGSGTDYTLTISS LQPEDFATYYCQQGKMLPWTFGQGTKVEIKRTVAAPSVF	308.72 mg/l	[15]
22	Thioredoxin from *E. coli*	pelB(A9E)	MKYLLPTAEAGLLLLLAAPQIASDKIIHLTDDSFDTDVLKADGAILVDFW AEWCGPCKMIAPILDEIADEYQGKLTVAKLNIDQNPGTAPKYGIRGIPTL LLFKNGEVAATKVGALSKGQLKEFLDANLA	20 mg/l	[16]

23	Thioredoxin from *E. coli*	malE(A14E)	MKIKTGARILALSELTTMMFSASALASDKIIHLTDDSFDTDVLKADGAILV DFWAEWCGPCKMIAPILDEIADEYQGKLTVAKLNIDQNPGTAPKYGIRG IPTLLLFKNGEVAATKVGALSKGQLKEFLDANLA	0	[16]
24	beta-mannanase from *Bacillus licheniformis* (mannan endo-1,4-beta-mannosidase)	OmpA	MKKTAIAIAVALAGFATVAQASEANGAALSNPNANQTTKNVYSWLANL PNKSNKRVVSGHFGGYSDSTLAWIKQCARELTGKMPGILSCDYKNWQ TRLYVADQISYGCNQELINFWNQGGLVTISVHMPNPGFHSGENYKTIL PT SQFQNLTNHRTTEGRRWKDMLDKMADGLDELQNNGVTVLFRPLHEM NGEWFWWGAEGYNQFDQTRANAYISAWRDMYQYFTHERKLNNLIWV YSPDVYRDHVTSYYPGANYVDIVALDSYHPDPHSLTDQYNRMIALDKP FAFAEIGPPESMAGSFDYSNYIQAIKQKYPRTVYFLAWNDKWSPHNNR GAWDLFNDSWVVNRGEIDYGQSNPATVLYDFENNTLSWSGCEFTDG GPWTSNEWSANGTQSLKADVVLGNNSYHLQKTVNRNLSSFKNLEIKV SHSSWGNVGSGMTARVFVKTGSAWRWNAGEFCQFAGKRTTALSIDL TKVSNLHDVREIGVEYKAPANSNGKTAIYLDHVTVRHHHHHH	44.5 mg/l	[17]
25	Chicken interferon beta (IFN-beta)	nil	MGSSHHHHHHSSGLVPRGSHMASMTGGQQMGRGSEFCNHLRHQD ANFSWKSLQLLQNTAPPPPQPCPQQDVTFPFPETLLKSKDKKQAAITT LRILQHLFNMLSSPHTPKHWIDRTRHSLLNQIQHYIHHLEQCFVNQGTR SQRRGPRNAHLSINKYFRSIHNFLQHNNYSACTWDHVRLQARDCFRH VDTLIQWMKSRAPLTASSKRLNTQHHHHHH	0	[18]
26	Glutaminase from *Bacillus licheniformis* DSM13	ompA	MKKTAIAIAVALAGFATVAQAMNEVLEERYDARFWQSRLEDLVEHYRP FSSSGRNAEYIPALGKIDSNQLGICVIGSDQTMIKAGNSDVSFTLQSISK VISFIAACLTKGISYVLDRVDVEPTGDAFNSIIRLEMHKPGKPFNPMINA GALTVSSILPGESALGKIESLHDVIEKMIGKRLEINEEVFRSEWQTAHRN RALAHYLKETGFLEADVEETLEVYLKQCSMEGSTEDIALIGMILANDGY HPFRREHVIPKDVARLTKALMLTCGMYNASGKFAAFVGIPAKSGVSGG IMCAVPASVKREQPFQHGCGIGIYGPAIDDYGNSMTGGMLLKHIAREW DLSIFHHHHHHHHHHLDYKDDDDK	80 mg/l	[19]

27	Falcipain-2 from human malaria parasite	nil	MGSHHHHHHKELNRFADLTYHEFKNKYLSLRSSKPLKNSKYLLDQMN YEEVIKKYRGEENFDHAAYDWRLHSGVTPVKDQKNCGSCWAFSSIGS VESQYAIRKNKLITLSEQELVDCSFKNYGCNGGLINNAFEDMIELGGICP DGDYPYVSDAPNLCNIDRCTEKYGIKNYLSVPDNKLKEALRFLGPISISV AVSDDFAFYKEGIFDGECGDQLNHAVMLVGFGMKEIVNPLTKKGEKHY YYIIKNSWGQQWGERGFINIETDESGLMRKCGLGTDAFIPLIE	0	[20]
28	human N-terminal domain of T1R3 taste receptor (hT1R3-NTD)	nil	MGSSHHHHHHSSGLVPRGSHMAPLCLSQQLRMKGDYVLGGLFPLGE AEEAGLRSRTRPSSPVCTRFSSNGLLWALAMKMAVEEINNKSDLLPGL RLGYDLFDTCSEPVVAMKPSLMFLAKAGSRDIAAYCNYTQYQPRVLAV IGPHSSELAMVTGKFFSFFLMPQVSYGASMELLSARETFPSFFRTVPS DRVQLTAAAELLQEFGWNWVAALGSDDEYGRQGLSIFSALAAARGICI AHEGLVPLPRADDSRLGKVQDVLHQVNQSSVQVVLLFASVHAAHALF NYSISSRLSPKVWVASEAWLTSDLVMGLPGMAQMGTVLGFLQRGAQL HEFPQYVKTHLALATDPAFCSALGEREQGLEEDVVGQRCPQCDCITL QNVSAGLNHHQTFSVYAAVYSVAQALHNTLQCNASGCPAQDPVKPW QLLENMYNLTFHVGGLPLRFDSSGNVDMEYDLKLWVWQGSVPRLHD VGRFNGSLRTERLKIRWHTSDNQKPVSSAWSHPQFEK	0	[21]
29	Recombinant human monokine induced by IFN-gamma (rHuMig)	nil	MATPVVRKGRCSCISTNQGTIHLQSLKDLKQFAPSPSCEKIEIIATLKNG VQTCLNPDSADVKELIKKWEKQVSQKKKQKNGKKHQKKKVLKVRKSQ RSRQKKTT	0	[22]
30	Phospholipase A2 (PLA_2) from *Streptomyces violaceoruber*	pelB	MKYLLPTAAAGLLLLAAQPAMAAPADKPQVLASFTQTSASSQNAWLAA NRNQSAWAAYEFDWSTDLCTQAPDNPFGFPFNTACARHDFGYRNYK AAGSFDANKSRIDSAFYEDMKRVCTGYTGEKNTACNSTAWTYYQAVK IFG	0	[23]

31	N-terminus domain of *Pinellia ternata*	alkaline phosphatase (APSP)	MVKQSTIALALLPLLFTPVTKAAVGTNHLLSGEILDTNGHLRNGDFDLV MQEDCNAVLYNGNWQSNTANKGRDCKLTLTNRGELIIKNGDGSIVFRS GSQSERGDYALVVHPEGKLVIYGPSVFEINPWVPGLEHHHHHH	20 mg/l	[24]
32	Valencene dioxygenase (ValOx) from *Pleurotus sapidus*	gIII	MKKLLFAIPLVVPFYSHSTMELEMVHNISLSSRKALHNVHLPYMVQLPK PTGYNVALKNAAEGYDKARRMVAWLYDIADYESSIPQTFTLQQKTDKY TWELSDNFPPHLAVVPPDQSVSAPSIFSPVRLAQTLLIMSSLWYDDHT DLAPGPEQNTMQKLTQWNQERHKDQGWLIKDMFNAPNIGLRNDWYT DEVFAQQFFTGPNSTTITLASDVWLTAFTSEAKAQGKDKVIALFESAPP NSFYVQDFSDFRRRMGAKPDEELFNDSDGAMRYGCAAVALFYLTAM GKLHPLAIIPDYKGSMAASVTIFNKRTNPLDISVNQANDWPWRYAKTCV LSSDWALHEMIIHLNNTHLVEEAVIVAAQRKLSPSHIVFRLLEPHWVVTL SLNALARSVLIPEVIVPIAGFSAPHIFQFIRESFTNFDWKSLYVPADLESR GFPVDQLNSPKFHNYAYARDINDMWTTLKKFVSSVLQDAQYYPDDAS VAGDTQIQAWCDEMRSGMGAGMTNFPESITTVDDLVNMVTMCIHIAA PQHTAVNYLQQYYQTFVPNKPSALFSPLPTSIAQLQKYTESDLMAALP LNAKRQWLLMAQIPYLLSMQVQEDENIVTYAANASTDKDPIIASAGRQL AADLKKLAAVFLVNSAQLDDQNTPYDVLAPEQLANAIVIHHHHHH	0	[25]
33	5S-scFv	pelB	MKYLLPTAAAGLLLLAAQPAMAMAEVQLVQSGAEVAKPGASVKVSCK ASGYSFSTYNIHWVRQAPGQGLEWIGTIYPGIGDTSYNQKFKGKATLT ADKSTSTAYLELSSLRSEDTAVYYCARSDIYYGNYNALDYWGQGTLVT VSSSGGGSGGGGTGGGGSIVMTQSPLSLPVTPGEPASISCRASQSIV HSYGDTYLEWYLQKPGQSPQLLIYKVSNRFSGVPDRFSGSGSGTDFT LKISRVEAEDVGVYYCFQRSYVPWTFGQGTKVEIKRAAALEHHHHHH	10 mg/l	[26]

34	Lupanine hydroxylase from *Pseudomonas* sp.	native	MSANKNIWIIRLGVAFVCVAIGAAQANEKDGSAVTSGNWSLLGGGNEQ HYFSALKDVNKSNVKNLGLSWFTDMEAGDGLVGNPLVADGVIYQGGP PGKIYANDLKTGKNLWTYTPEVQYDKDTSWTGFWFTHVNRGLAVDDD NVYIGSYCKLLAVSRTTHKLTWSSQSCDPKKMQAITGAPRVGGGKVFI GNASGDFGGDRGHLDAFDAKTGKHLWRFYTMPGDPSKPFENDLLAK ASKTWGTDYWKYTKGGVSPWDAITYDEASDTLYFGTDGPSPWSPAQ RAPDAGDELFSHSIIAVDASTGAYKWHFQTVQNDGSNMSATMHIMLA DLPVEGVSKRVVMTAPKNGYFYVLDASTGKFISADHYVPVNWTKGLD PKTGRPIPSNEANYWERPGEMTIPLPGDVGGHNWEAMAYNPELRTVY IPSTLVPVTVVASKDTGELDLDYYYGMRPDATIKTQGDLVAWDPLLQK EKWRAKRSLPVNGGVLATAGGLVFQGTGDGHFEAFDANTGEKLWSF HVGGSILAAPTTVEVDGDQYLIVASGNGGASGMRGIPRLMNNLQSQG PARLLAFRLGGKTELPITSTPDFPKPQYPKPTSAMAESGRHIFNANACG ACHGFNAEGSTPGLPDLRRSDKLDLAVMKSIVIDGAFKPLGMPGHPHI SDADLQALQAFILQKAWTAYDTQQTLKTSDTGAQ	5.2 mg/l	[27]
35	Shiga toxin 2 subunit A	native	MKCILFKWVLCLLLGFSSVSYSREFTIDFSTQQSYVSSLNSIRTEISTPL EHISQGTTSVSVINHTPPGSYFAVDIRGLDVYQARFDHLRLIIEQNNLYV AGFVNTATNTFYRFSDFTHISVPGVTTVSMTTDSSYTTLQRVAALERS GMQISRHSLVSSYLALMEFSGNTMTRDASRAVLRFVTVTAEALRFRQI QREFRQALSETAPVYTMTPGDVDLTLNWGRISNVLPEYRGEDGVRVG RISFNNISAILGTVAVILNCHHQGARSVRAVNEDSQPECQITGDRPVIKI NNTLWESNTAAAFLNRKSQFLYTTGK	3.5 mg/l	[28]
36	Shiga toxin 2 subunit B	native	MKKMFMAVLFALASVNAMAADCAKGKIEFSKYNENDTFTVKVAGKEY WTSRWNLQPLLQSAQLTGMTVTIKSSTHHHHHH	3.5 mg/l	[28]
37	cystatin-glutathione S-transferase (GST) from *S. japonicum*	ompA	MKKTAIAIAVALAGFATVAQAGAPVPVDENDEGLQRALQFAIAEYNRAS NDKYSSRVVRVISAKRQLVSGIKYILQVEIGRTTCPKSSGDLQSCEFHD EPELAKYTTCTFVVYSIPWLNQIKLLESKCQGGGGGMSPILGYWKIKGL VQPTRLLLETLEEKYEEHLYERDEGDKWRNKKFELGLEFPNLPYYIDG DVKLTQSMAIIRYIADKHNMLGGSPKERAEISMLEGAVLDIRYGVSRIAY SKDFETLKVDFLSKLPEMLKMFEDRLSHKTYLNGDHVTHPDFMLYDAL DVVLYMDPMCLDAFPKLVSFKKRIEAIPQIDKYLKSSKYIAWPLQGWQA TFGGGDHPPK	0.35 mg/l	[29]

38	alpha-amylase from *Streptomyces thermoviolaceus*	native	MASRTLSGALALAAAATAVLAAPATVAHRSPPGTKDVTAVLFEWDYVS<br>VAKECTSTLGPAGYGYVQVSPPAEHIQGSQWWTSYQPVSYKIAGRLG<br>DRAAFRSMVNTCHAAGVKVVVDTVINHMSAGSGTGTGGSSYTKYDYP<br>GLYSAPDFDDCTAEITDYQDRWNVQHCELVGLADLDTGEEYVRQTIA<br>GYMNDLLSLGVDGFRIDAATHIPAEDLANIKSRLSNPNAYWKQEVIYGA<br>GEPPKPGEYTGTGDVQEFRYAYDLKRVFTQEHLAYLKNYGEDWGYLS<br>STTAGVFVDNHDTERNGSTLNYKNDATYTLANVFMLAWPYGAPDINS<br>GYEWSDPDAGPPDGGHVDACWQNGWKCQHKWPEIASMVAFRNATR<br>GEPVTDWWDDGADAIAFGRGSKGFVAINHESATVQRTYQTSLPAGTY<br>CDVQSNTTVTVDSAGRFTAALGPDTALALHNGRTSC	281 mg/l	[30]
39	Exotoxin A from *Pseudomonas aeruginosa*	ompA	MKKTAIAIAVALAGFATVAQAAEEAFDLWNECAKACVLDLKDGVRSSR<br>MSVDPAIADTNGQGVLHYSMVLEGGNDALKLAIDNALSITSDGLTIRLE<br>GGVEPNKPVRYSYTRQARGSWSLNWLVPIGHEKPSNIKVFIHELNAGN<br>QLSHMSPIYTIEMGDELLAKLARDATFFVRAHESNEMQPTLAISHAGVS<br>VVMAQAQPRREKRWSEWASGKVLCLLDPLDGVYNYLAQQRCNLDDT<br>WEGKIYRVLAGNPAKHDLDIKPTVISHRLHFPEGGSLAALTAHQACHLP<br>LETFTRHRQPRGWEQLEQCGYPVQRLVALYLAARLSWNQVDQVIRNA<br>LASPGSGGDLGEAIREQPEQARLALTLAAAESERFVRQGTGNDEAGA<br>ANADVVSLTCPVAAGECAGPADSGDALLERNYPTGAEFLGDGGDVSF<br>STRGTQNWTVERLLQAHRQLEERGYVFVGYHGTFLEAAQSIVFGGVR<br>ARSQDLDAIWRGFYIAGDPALAYGYAQDQEPDARGRIRNGALLRVYVP<br>RSSLPGFYRTSLTLAAPEAAGEVERLIGHPLPLRLDAITGPEEEGGRLTI<br>LGWPLAERTVVIPSAIPTDPRNVGGDLDPSSIPDKEQAISALPDYASQP<br>GKPPREDLK	60 mg/l	[31]
40	Diphtheria toxin	unknown	MDPSRKLFASILIGALLGIGAPPSAHAGADDVVDSSKSFVMENFSSYH<br>GTKPGYVDSIQKGIQKPKSGTQGNTDDDWKEFYSTDNKYDAAGYSVD<br>NENPLSGKAGGVVKVTYPGLTKVLALKVDNAETIKKELGLSLTEPLME<br>QVGTEEFIKRFGDGASRVVLSLPFAEGSSSVETINNWEQAKALSVELEI<br>NFETRGKRGQDAMYEYMAQACAGNRVRRSPGIRNHGHSCFLCEIVIR<br>SQFHTTYEPEAGPIKNKMSESPNKTVSEEKAKQYLEEFHQTALEHPEL<br>SELKTVTGTNPVFAGANYAAWAVNVAQVIDSETADNLEKTTAALSILPG<br>IGSVMGIADGAVHHNTEEIVAQSIALSSLMVAQAIPLVGELVDIGFAAYN<br>FVESIINLFQVVHNSYNRPAYSPGHKTQPFLHDGYAVSWNTVEDSIIRT<br>GFQGESGHDIKITAENTPLPIAGVLLPTIPGKLDVNKSKTHISVNGRKIR<br>MRCRAIDGDVTFCRPKSPVYVGNGVHAAAASYSMEHFRWGKPV	0.7 mg/l	[32]

41	human epidermal growth factor (hEGF)	phoA	MKQSTIALALLPLLFTPVTKANSDSECPLSHDGYCLHDGVCMYIEALDK YACNCVVGYIGERCQYRDLKWWELR	1.026 mg/l	[33]
42	Creatinase from *Pseudomonas putida*	Chitinase from *Aeromonas hydrophila*	MLSPKLSLLALLVGGLCTTSAFAMQMPKTIQIKNGEKVKPTFSQQEYA NRQSKLRTYLAQNNIDAAVFTSYHNINYYSDFLYCSFGRPYALVVTQEA VVSISANIDGGQPWRRTVGTDNIIYTDWQRDNYFVAIQQALPKAGRIGI EFDHLNLMNRDKLASRYPQAELVDIAAPCMRMRMIKSAEEHAIIRQGA RVADIGGAAVVEALRDQVPEYEVALHATQAMVREIARTYPDSELMDT WTWFQSGINTDGAHNPVTSRKVNKGDILSLNCFPMIAGYYTALERTLF LDHCSDEHLRLWEVNVKVHEAGLELVKPGMRSSDIALQLNEIFLEHDL LQYRTFGYGHSFGTLSHYYGREAGLELREDIDTVLEPGMVVSIEPMIML PEGLPGAGGYREHDILIVNEHGSENITKFPYGPEHNIIKK	479.45 mg/l	[34]
43	Human protein disulfide isomerase (hPDI)	Unknown (modified from ompA)	MKKTAIAIAVALAGFATVAQADAPEEEDHVLVLRKSNFAEALAAHKYLL VEFYAPWCGHCKALAPEYAKAAGKLKAEGSEIRLAKVDATEESDLAQ QYGVRGYPTIKFFRNGDTASPKEYTAGREADDIVNWLKKRTGPAATTL PDGAAAESLVESSEVAVIGFFKDVESDSAKQFLQAAEAIDDIPFGITSNS DVFSKYQLDKDGVVLFKKFDEGRNNFEGEVTKENLLDFIKHNQLPLVIE FTEQTAPKIFGGEIKTHILLFLPKSVSDYDGKLSNFKTAAESFKGKILFIFI DSDHTDNQRILEFFGLKKEECPAVRLITLEEEMTKYKPESEELTAERITE FCHRFLEGKIKPHLMSQELPEDWDKQPVKVLVGKNFEDVAFDEKKNV FVEFYAPWCGHCKQLAPIWDKLGETYKDHENIVIAKMDSTANEVEAVK VHSFPTLKFFPASADRTVIDYNGERTLDGFKKFLESGGQDGAGDDDDL EDLEEAEEPDMEEDDDQKAVKDEL	30 mg/l	[35]
44	V_HHs A4.2	ompA	MKKTAIAIAVALAGFATVAQAQVKLEESGGGLVQAGGSLRLSCAASGR TFNTLSMGWFRQAPGKEREFVAAVSRSGGSTYYADSVKGRFTVSRD NAKKTVYLQMNSLKPEDTAVYYCAAAATKSNTTAYRLSFDYWGQGTQ VTVSSEQKLISEEDLHHHHHH	31.3 mg/l	[36]
45	V_HHs A5.1	ompA	MKKTAIAIAVALAGFATVAQAQVKLEESGGGLVQAGGSLRLSCAASGR TFSMYRMGWFRQAPGKEREFVAVITRNGSSTYYADSVKGRFTISRDN AKKTVYLQMNSLKPEDTALYYCAATSGSSYLDAAHVYDYWGQGTQVT VSSEQKLISEEDLHHHHHH	55.5 mg/l	[36]

46	V_HHs A19.2	ompA	MKKTAIAIAVALAGFATVAQAQVKLEESGGGLVQPGGSLRLSCAASGRTLSSYIVAWFRQPPGKEREFVAGIISRRGGNSAYVESVKGRFTISRDNAKKTVYLQMNSLKPEDTAVYYCAADGSVAGWGRRSVSVSSYDYWGQGTQVTVSSEQKLISEEDLHHHHHH	3.8 mg/l	[36]
47	V_HHs A20.1	ompA	MKKTAIAIAVALAGFATVAQAQVQLVESGGGLAQAGGSLRLSCAASGRTFSMDPMAWFRQPPGKEREFVAAGSSTGRTTYYADSVKGRFTISRDNAKKTVYLQMNSLKPEDTAVYYCAAAPYGANWYRDEYDYWGQGTQVTVSSEQKLISEEDLHHHHHH	72.3 mg/l	[36]
48	V_HHs A24.1	ompA	MKKTAIAIAVALAGFATVAQAQVQLVESGGGLVQAGGSLRLSCAASIRSFSNRNMGWFRQPPGKEREFVAGISWGGGSTRYADSVKGRFTISRDNAKKTVYLQMNSLKPEDTAVYYCAAEFGHNIATSSDEYDYWGQGTQVTVSSEQKLISEEDLHHHHHH	8.5 mg/l	[36]
49	V_HHs A26.8	ompA	MKKTAIAIAVALAGFATVAQAQVKLEESGGGLVQAGGSLRLSCAASERTFSRYPVAWFRQAPGAEREFVAVISSTGTSTYYADSVKGRFTISRDNAKVTVYLQMNNLKREDTAVYFCAVNSQRTRLQDPNEYDYWGQGTQVTVSSEQKLISEEDLHHHHHH	64.9 mg/l	[36]
50	V_HHs B5.2	ompA	MKKTAIAIAVALAGFATVAQAQVQLVESGGGLVQPGGSLRLSCAASGNIFSINTMGWYRQAPGKQLELVAAITSGGTTSYTDSVEGRFTISRDNAKNAVYLQMNSLKAEDTAVYYCNTVKVVGGRLDNPDYWGQGTQVTVSSEQKLISEEDLHHHHHH	6.7 mg/l	[36]
51	V_HHs B7.3	ompA	MKKTAIAIAVALAGFATVAQAQVKLEESGGGLVQPGGSLRLSCAASGRTASGYGMGWFRQAPGKEREFVAAISRSGAGTLNADFVKGRFTISRDNAKNTVYLQMNSLKPEDTAVYYCVARPTKVDRDYATRREMYNYWGQGTQVTVSSEQKLISEEDLHHHHHH	1.5 mg/l	[36]
52	V_HHs B13.2	ompA	MKKTAIAIAVALAGFATVAQAQVKLEESGGGSVQAGGSLRLSCAASGRDFSTLAMGWFRQAPGKEREFVATINWSGGTTHYADSVKGRFTISRDNAKNTVYLQMGSLKPEDTAVYYCGRSKYAAGALTRAYDYNYWGQGTQVTVSSEQKLISEEDLHHHHHH	4.0 mg/l	[36]

53	V_HHs B13.3	ompA	MKKTAIAIAVALAGFATVAQAQVKLEESGGGLVQAGGSLRLSCSASGSIFSINDMGWYRRAPGKRRELVAAITSGGIPNYADSVKGRFTISRDNAKNTGYLQMNSLKPEDTAVYYCAAQFGTVAAALRRHEYDYWGQGTQVTVSSEQKLISEEDLHHHHHH	1.6 mg/l	[36]
54	V_HHs B13.6	ompA	MKKTAIAIAVALAGFATVAQAQVKLEESGGGLVQAGGSLRLSCSASGRTFSSGVMGWFRQAPGKQRELVAAITTGGSTSYTDSVKGRFTISRDNAKNTVYLQMNSLKPEDTAVYYCNSVAVVGGVIKSPDYWGQGTQVTVSSEQKLISEEDLHHHHHH	3.6 mg/l	[36]
55	V_HHs B15.3	ompA	MKKTAIAIAVALAGFATVAQAQVQLVESGGGSVQAGGSLRLSCAASGLSRYAMAWFRQGTGKEREFVASTNWSSGNTPYADSVKGRFIISRDNAKNTVYLQMNSLKPGDTAIYYCAARKLDVPSRYSQHYDYWGQGTQVTVSSEQKLISEEDLHHHHHH	1.2 mg/l	[36]
56	V_HHs B15.5	ompA	MKKTAIAIAVALAGFATVAQAQVQLVESGGDLVQAGGSLRLSCAASGSISRISTMGWYRQAPGKQRELVATISTGGTTNYAESVKGRFTVSRDNAKNTMYLQMNSLKPEDTAVYYCAAGWKVVRGSLEYEYSGQGTQVTVSSEQKLISEEDLHHHHHH	4.6 mg/l	[36]
57	Immunotoxins	PelB	MKYLLPTAAAGLLLLAAQPAMA*HHLGGAKQAGNV*QVKLQESGTELAKPGAAVKMSCKASGYTFTDYWMHWVKQRPGQGLEWIGYINPNTAYTDYNQKFKDKATLTADKSSSTAYMQLRSLTSEDSAVYYCAKKTTQTTWGFPFPFWGQGTTVTVSSGGGGSGGGGSGGGGSDIVLTQSPKSMAMSVGERVTLSCKASENVDSFVSWYQQKPGQSPKLLIYGASNRYTGVPDRFAGSGSGRDFTLTISSVQAEDLADYHCGQNYRYPLTFGAGTKLEIKREGGSLAALTAHQACHLPLETFTRHRQPRGWEQLEQCGYPVQRLVALYLAARLSWNQVDQVIRNALASPGSGGDLGEAIREQPEQARLALTLAAAESERFVRQGTGNDEAGAASADVVSLTCPVAAGECAGPADSGDALLERNYPTGAEFLGDGGDVSFSTRGTQNWTVERLLQAHRQLEERGYVFVGYHGTFLEAAQSIVFGGVRARSQDLDAIWRGFYIAGDPELAYGYAQDQEPDARGRIRNGALLRVYVPRSSLPGFYRTGLTLAAPEAAGEVERLIGHPLPLRLDAITGPEEEGGRLETILGWPLAERTVVIPSAIPTDPRNVGGDLDPSSIPDKEQAISALPDYASQPGKPPREDLK	0.6 g/l	[37]

58	Green fluorescent protein (GFP)	TorA	MANNNDLFQASRRRFLAQLGGLTVAGMLGPSLLTPRRATAAQA*XXXX* RKGEELFTGVVPILVELDGDVNGHKFSVSGEGEGDATYGKLTLKFICTT GKLPVPWPTLVTTFGYGVQCFARYPDHMKQHDFFKSAMPEGYVQER TIFFKDDGNYKTRAEVKFEGDTLVNRIELKGIDFKEDGNILGHKLEYNYN SHNVYIMADKQKNGIKVNFKIRHNIEDGSVQLADHYQQNTPIGDGPVLL PDNHYLSTQSALSKDPNEKRDHMVLLEFVTAAGITHGMDELYK	7.5 mg/l	[38]
59	20-kDa human growth hormone (20K hGH)	*npr*	MGLGKKLSSAVAASFMSLTISLPGVQAFPTIPLSRLFDNASLRAHRLHQ LAFDTYQEFEEAYIPKEQKYSFLQNPQTSLCFSQSIPTPSNREETQQKS NLELLRISLLLIQSWLEPVQFLRSVFANSLVYGASDSNVYDLLKDLEEGI QTLMGRLEDGSPRTGQIFKQTYSKFDTNSHNDDALLKNYGLLYCFRKD MDKVETFLRIVQCRSVEGSCGF	9.8 mg/l	[39]
60	Human interferon-gamma	modified penicillin acylase signal peptide	MKNRNRMIVNCVTASLMYYSSLPALAQDPYVKEAENLKKYFNAGHSD VADNGTLFLGILKNWKEESDRKIMQSQIVSFYFKLFKNFKDDQSIQKSV ETIKEDMNVKFFNSNKKKRDDFEKLTNYSVTDLNVQRKAIHELIQVMAE LSPAAKTGKRKRSQMLFRGRRASQ	2.2 mg/l	[40]
61	Human interleukin-2 (hIL-2)	modified penicillin acylase signal peptide	MKNRNRMIVNCVTASLMYYSSLPALAAPTSSSTKKTQLQLEHLLLDLQ MILNGINNYKNPKLTRMLTFKFYMPKKATELKHLQCLEEELKPLEEVLN LAQSKNFHLRPRDLISNINVIVLELKGSETTFMCEYADETATIVEFLNRW ITFCQSIISTLT	ND	[40]
62	Human growth hormone (hGH)	*ompA*	MKKTAIAIAVALAGFATVAQAFPTIPLSRLFDNAMLRAHRLHQLAFDTY QEFEEAYIPKEQKYSFLQNPQTSLCFSESIPTPSNREETQQKSNLELLR ISLLLIQSWLEPVQFLRSVFANSLVYGASDSNVYDLLKDLEEGIQTLMG RLEDGSPRTGQIFKQTYSKFDTNSHNDDALLKNYGLLYCFRKDMDKVE TFLRIVQCRSVEGSCGF	28.4 mg/l	[41]
63	Granulocyte-macrophage colony-stimulating	CSP	MKKKLLALALLALLFNGAQAPARSPSPSTQPWEHVNAIQEARRLLNLS RDTAAEMNETVEVISEMFDLQEPTCLQTRLELYKQGLRGSLTKLKGPL TMMASHYKQHCPPTPETSCATQIITFESFKENLKDFLLVIPFDCWEPVQ EGS*EQKLISEEDL*NS*HHHHHH*	0.8 g/l	[42]

	factor (GM-CSF)				
64	Human interferon-gamma	SP1	MAPSGKSTLLLLFLLLCLPSWNAGACYCQDPYVKEAENLKKYFNAGHSDVADNGTLFLGILKNWKEESDRKIMQSQIVSFYFKLFKNFKDDQSIQKSVETIKEDMNVKFFNSNKKKRDDFEKLTNYSVTDLNVQRKAIHELIQVMAELSPAAKTGKRKRSQMLFRGRRASQ	5 mg/l	[43]
65	beta-lactoglobulin (rBLG)	pelB	MKYLLPTAAAGLLLLAAQPAMA*MDIGINS*MLIVTQTMKGLDIQKVAGTWYSLAMAASDISLLDAQSAPLRVYVEELKPTPEGDLEILLQKWENGECAQKKIIAEKTKIPAVFKIDALNENKVLVLDTDYKKYLLFCMENSAEPEQSLACQCLVRTPEVDDEALEKFDKALKALPMHIRLSFNPTQLEEQCHI*LEHHHHHH*	24 ng/μg protein	[44]
66	Cellulose binding domain (CBD)	Cex	MDPRTTPAPGHPARGARTALRTTLAAAAATLVVGATVVLPAQAASSGPAGCQVLWGVNQWNTGFTANVTVKNTSSAPVDGWTLTFSFPSGQQVTQAWSSTVTQSGSAVTVRNAPWNGSIPAGGTAQFGFNGSHTGTNAAPTAFSLNGTPCTVG	5.31 g/l	[45]
67	Human insulin-like growth factor II (IGF-II)	SpA	MKKKNIYSIRKLGVGIASVTLGTLLISGGVTPAANAVDNKFNKEQQNAFYEILHLPNLNEEQRNAFIQSLKDDPSQSANLLAEAKKLNDAQAPKVDNKFNKEQQNAFYEILHLPNLNEEQRNAFIQSLKDDPSQSANLLAEAKKLNDAQAPKMAYRPSETLCGGELVDTLQFVCGDRGFYFSRPASRVSRRSRGIVEECCFRSCDLALLETYCATPAKSE	0.01 mg/l	[46]
68	Single-chain antibody Fv fragment (scFv)	ompA	MKKTAIAIAVALAGFATVAQADIELTQTTSSLSASLGDRVTISCRASQDISNYLNWYQQNPDGTVKLLIYYTSNLHSEVPSRFSGSGSGTDYSLTISNLEQEDIATYFCQQDFTLPFTFGGGTAA	0.182 mg/l	[47]

69	Single-chain antibody Fv fragment (scFv)	mBiP	MMKFTVVAAALLLLGAVRADIELTQTTSSLSASLGDRVTISCRASQDISNYLNWYQQNPDGTVKLLIYYTSNLHSEVPSRFSGSGSGTDYSLTISNLEQEDIATYFCQQDFTLPFTFGGGTAA	115 mg/l	[47]
70	Single-chain antibody Fv fragment (scFv)	yBGL2	MRFSTTLATAATLFFTASQVSADIELTQTTSSLSASLGDRVTISCRASQDISNYLNWYQQNPDGTVKLLIYYTSNLHSEVPSRFSGSGSGTDYSLTISNLEQEDIATYFCQQDFTLPFTFGGGTAA	0.045 mg/l	[47]
71	Single-chain antibody Fv fragment (scFv)	yHSP150	MQYKKTLVASALAATTLADIELTQTTSSLSASLGDRVTISCRASQDISNYLNWYQQNPDGTVKLLIYYTSNLHSEVPSRFSGSGSGTDYSLTISNLEQEDIATYFCQQDFTLPFTFGGGTAA	21 mg/l	[47]
72	single-chain antibody variable fragment with affinity for hapten 2-phenyloxazol-5-one (scFv-phOx)	pelB	MKYLLPTAAAGLLLLAAQPAMAQVQLVQSGGEVKKPGASVKVSCKASGYTFTSYGISWVRQAPGQGLEWMGWISAYNGNTKYAQKLQGRVTMTTDTSTSTAYMELRSLRSDDTAVYYCVRLLPKRTATLHYYIDVWGKGTLVTVSSGS*EQKLISEEDL*NS*HHHHHH*	0.3 mg/l	[48]
73	scFv specific for CD3 T cell surface antigen (scFv-dmOKT3)	pelB	MKYLLPTAAAGLLLLAAQPAMAQVQLQQSGAELARPGASVKMSCKASGYTFTRYTMHWVKQRPGQGLEWIGYINPSRGYTNYNQKFKDKATLTTDKSSSTAYMQLSSLTSEDSAVYYCARYYDDHYCLDYWGQGTTLTVSS*SEEGEFSEAREDMAALEKG*QIVLTQSPAIMSASPGEKVTMTCSASSSVSYMNWYQQKSGTSPKRWIYDTSKLASGVPAHFRGSGSGTSYSLTISGMEAEDAATYYCQQWSSNPFTFGSGTKLEING*SEQKLISEEDL*NS*HHHHHH*	0.2 mg/l	[48]

74	Human granulocyte-macrophage colony stimulating factor (HuGM-CSF)	ompA	MKKTAIAIAVALAGFATVAQAAPARSPSPSTQPWEHVNAIQEARRLLNL<br>SRDTAAEMNETVEVISEMFDLQEPTCLQTRLELYKQGLRGSLTKLKGP<br>LTMMASHTKQHCPPTPETSCATQIITFESFKENLKDFLLVIPFDCWEPV<br>QE	0.104 mg/l	[49]
75	Staphylokinase (SAK)	ompA	MKKTAIAIAVALAGFATVAQASSSFDKGKYKKGDDASYFEPTGPYLMV<br>NVTGVDGKGNELLSPHYVEFPIKPGTTLTKEKIEYYVEWALDATAYKEF<br>RVVELDPSAKIEVTYYDKNKKKEETKSFPITEKGFVVPDLSEHIKNPGF<br>NLITKVVIEKK	15 mg/l	[50]
76	Cytochrome P450	*phoA*	MKQSTIALALLPLLFTPVTKAMTESTTDPARQNLDPTSPAPATSFPQDR<br>GCPYHPPAGYAPLREGRPLSRVTLFDGRPVWAVTGHALARRLLADPR<br>LSTDRSHPDFPVPAERFAGAQRRRVALLGVDDPEHNTQRRMLIPTFSV<br>KRIGALRPRIQETVDRLLDAMERQGPPAELVSAFALPVPSMVICALLGV<br>PYADHAFFEERSQRLLRGPGADDVNRARDELEEYLGALIDRKRAEPG<br>DGLLDELIHRDHPDGPVDREQLVAFAVILLIAGHETTANMISLGTFTLLS<br>HPEQLAALRAGGTSTAVVVEELLRFLSIAEGLQRLATEDMEVDGATIRK<br>GEGVVFSTSLINRDADVFPRAETLDWDRPARHHLAFGFGVHQCLGQN<br>LARAELDIAMRTLFERLPGLRLAVPAHEIRHKPGDTIQGLLDLPVAW	600 nmol/ l<br><br>(27 mg/l equivalent)	[51]
77	Human granulocyte-colony stimulating factor (hG-CSF)	Endoxylanase from *Bacillus*	MFKFKKKFLVGLTAAFMSISMFSATASATPLGPASSLPQSFLLKCLEQV<br>RKIQGDGAALQEKLCATYKLCHPEELVLLGHSLGIPWAPLSSCPSQAL<br>QLAGCLSQLHSGLFLYQGLLQALEGISPELGPTLDTLQLDVADFATTIW<br>QQMEELGMAPALQPTQGAMPAFASAFQRRAGGVLVASHLQSFLEVS<br>YRVLRHLAQP	ND	[52]
78	Human leptin	Endoxylanase from *Bacillus*	MFKFKKKFLVGLTAAFMSISMFSATASAVPIQKVQDDTKTLIKTIVTRIND<br>ISHTQSVSAKQRVTGLDFIPGLHPILSLSKMDQTLAVYQQVLTSLPSQN<br>VLQIANDLENLRDLLHLLAFSKSCSLPQTSGLQKPESLDGVLEASLYST<br>EVVALSRLQGSLQDILQQLDVSPEC	150 mg/l	[53]

79	Human leptin (hOB)	ompA	MKKTAIAIAVALAGFATVAQAMHWGTLCGFLWLWPYLFYVQAVPIQKVQDDTKTLIKTIVTRINDISHTQSVSSKQKVTGLDFIPGLHPILTLSKMDQTLAVYQQILTSMPSRNVIQISNDLENLRDLLHVLAFSKSCHLPWASGLETLDSLGGVLEASGYSTEVVALSRLQGSLQDMLWQLDLSPGC	122.5 mg/l	[54]
80	Human granulocyte-colony stimulating factor (hG-CSF)	pelB	MKYLLPTAAAGLLLLAAQPAMAMTPLGPASSLPQSFLLKCLEQVRKIQGDGAALQEKLCATYKLCHPEELVLLGHSLGIPWAPLSSCPSQALQLAGCLSQLHSGLFLYQGLLQALEGISPELGPTLDTLQLDVADFATTIWQQMEELGMAPALQPTQGAMPAFASAFQRRAGGVLVASHLQSFLEVSYRVLRHLAQP*DPNSSSVDKLAAALEHHHHHH*	ND	[55]
81	Alkaline phosphatase	Endoxylanase from *Bacillus*	MFKFKKKFLVGLTAAFMSISMFSATASA*LQ*RTPEMPVLENRAAQGDITAPGGARRLTGDQTAALRDSLSDKPAKNIILLIGDGMGDSEITAARNYAEGAGGFFKGIDALPLTGQYTHYALNKKTGKPDYVTDSAASATAWSTGVKTYNGALGVDIHEKDHPTILEMAKAAGLATGNVSTAELQDATPAALVAHVTSRKCYGPSATSEKCPGNALEKGGKGSITEQLLNARADVTLGGGAKTFAETATAGEWQGKTLREQAQARGYQLVSDAASLNSVTEANQQKPLLGLFADGNMPVRWLGPKATYHGNIDKPAVTCTPNPQRNDSVPTLAQMTDKAIELLSKNEKGFFLQVEGASIDKQDHAANPCGQIGETVDLDEAVQRALEFAKKEGNTLVIVTADHAHASQIVAPDTKAPGLTQALNTKDGAVMVMSYGNSEEDSQEHTGSQLRIAAYGPHAANVVGLTDQTDLFYTMKAALGLK	5200 mg/l	[56]
82	Hirudin (HV1 variant)	ompA	MKKTAIVIAVALAGFATVAQAVVYTDCTESGQNLCLCEGSNVCGQGNKCILGSDGEKNQCVTGEGTPKPQSHNDGDFEEIPEEYLQ	300 mg/l	[57]
83	Fab' light chain	heat inducible enterotoxin II (stII)	MKKNIAFLLASMFVFSIATNAYADIQMTQSPSSLSASVGDRVTITCRASQDVNTAVAWYQQKPGKAPKLLIYSASFLYSGVPSRFSGSRSGTDFTLTISSLQPEDFATYYCQQHYTTPPTFGQGTKVEIKRTVAAPSVFIFPPSDEQLKSGTASVVCLLNNFYPREAKVQWKVDNALQSGNSQESVTEQDSKDSTYSLSSTLTLSKADYEKHKVYACEVTHQGLSSPVTKSFNRGEC	2 g/l	[58]

84	Fab’ heavy chain	heat inducible enterotoxin II (stII)	MKKNIAFLLASMFVFSIATNAYAEVQLVESGGGLVQPGGSLRLSCAAS GFNIKDTYIHWVRQAPGKGLEWVARIYPTNGYTRYADSVKGRFTISAD TSKNTAYLQMNSLRAEDTAVYYCSRWGGDGFYAMDYWGQGTLVTVS SASTKGPSVFPLAPSSKSTSGGTAALGCLVKDYFPEPVTVSWNSGALT SGVHTFPAVLQSSGLYSLSSVVTVPSSSLGTQTYICNVNHKPSNTKVD KKVEPKSCDKTHTCAA	2 g/l	[58]
85	enzymatically active version of tissue plasminogen activator (vtPA)	stII	MKKNIAFLLASMFVFSIATNAYACMHCSGENYDGKISKTMSGLECQAW DSQSPHAHGYIPSKFPNKNLKKNYCRNPDRELRPWCFTTDPNKRWEL CDIPRCVVGGCVAHPHSWPWQVSLRTRFGMHFCGGTLISPEWVLTAA HCLEKSPRPSSYKVILGAHQEVNLEPHVQEIEVSRLFLEPTRKDIALLKL SSPAVITDKVIPACLPSPNYVVADRTECFITGWGETQGTFGAGLLKEAQ LPVIENKVCNRYEFLNGRVQSTELCAGHLAGGTDSCQGDSGGPLVCF EKDKYILQGVTSWGLGCARPNKPGVYVRVSRFVTWIE*HHHHHH*	1409 ng/g cells (0.159 mg/l equivalent)	[59]
86	Human cytochrome P4501A1 (CYP1A1)	phoA	MKQSTIALALLPLLFTPVTKAMPSMYGLPAFVSATELLLAVTVFCLGFW VVRATRTWVPKGLKTPPGPWGLPFIGHMLTVGKNPHLSLTRLSQQYG DVLQIRIGSTPVVVLSGLNTIKQALVRQGDDFKGRPDLYSFTLITNGKS MTFNPDSGPVWAARRRLAQNALKSFSIASDPTSASSCYLEEHVSKEA NYLVSKLQKVMAEVGHFDPYKYLVVSVANVICAICFGQRYDHDDQELL SIVNLSNEFGEVTGSGYPADFIPVLRYLPNSSLDAFKDLNDKFYSFMKK LIKEHYRTFEKGHIRDITDSLIEHCQDRKLDENANVQLSDDKVITIVLDLF GAGFDTVTTAISWSLMYLVTNPRVQRKIQEELDTVIGRDRQPRLSDRP QLPYLEAFILETFRHSSFVPFTIPHSTTRDTSLNGFYIPKGCCVFVNQW QVNHDRELWGDPNEFRPERFLTPSGTLDKRLSEKVTLFGLGKRKCIGE TIGRSEVFLFLAILLQQIEFKVSPGEKVDMTPTYGLTLKHARCEHFQVQ MRSSGPQHLQA	25 nmol/l<br><br>(1.4 mg/l equivalent)	[60]
87	Cholera toxin B subunit (CT-B)	LTIIb-B	MSFKKIIKAFVIMAALVSVQAHAGPQNITDLCAEYHNTQIHTLNDKIFSYT ESLAGKREMAIITFKNGATFQVEVPGSQHIDSQKKAIERMKDTLRIAYLT EAKVEKLCVWNNKTPHAIAAISMAN	190 mg/l	[61]

88	Peptide:*N*-glycosidase F (PNGase F)	ompA	MKKTAIAIAVALAGFATVAQAAPADNTVNIKTFDKVKNAFGDGLSQSAEGTFTFPADVTAVKTIKMFIKNECPNKTCDEWDRYANVYVKNKTTGEWYEIGRFITPYWVGTEKLPRGLEIDVTDFKSLLSGNTELKIYTETWLAKGREYSVDFDIVYGTPDYKYSAVVPVVQYNKSSIDGVPYGKAHTLALKKNIQLPTNTEKAYLRTTISGWGHAKPYDAGSRGCAEWCFRTHTIAINNSNTFQHQLGALGCSANPINNQSPGNWTPDRAGWCPGMAVPTRIDVLNNSLIGSTFSYEYKFQNWTNNGTNGDAFYAISSFVIAKSNTPISAPVVTN*HHHHHH*	8 mg/l	[62]
89	Human proinsulin	DsbA	MKKIWLALAGLVLAFSASAAQYEDGKQYTTLEKPVAGAPQVLEFFSFFCPHCYQFEEVLHISDNVKKKLPEGVKMTKYHVNFMGGDLGKDLTQAWAVAMALGVEDKVTVPLFEGVQKTQTIRSASDIRDVFINAGIKGEEYDAAWNSFVVKSLVAQQEKAAADVQLRGVPAMFVNGKYQLNPQGMDTSNMDVFVQQYADTVKYLSEKK*GGGGGR*FVNQHLCGSHLVEALYLVCGERGFFYTPKTRREAEDLQVGQVELGGGPGAGSLQPLALEGSLQKRGIVEQCCTSICSLYQLENYCN	9.2 mg/g dry cell or 1.1 mg/l	[63]
90	native tissue-type plasminogen activator variant (rPA)	PelB	MKYLLPTAAAGLLLLAAQPAMASYQGNADCYFGNGSAYRGTHSLTESGASCLPWNSMILIGKVYTAQNPSAQALGLGKHNYCRNPDGDAKPWCHVLKNRRLTWEYCDVPSCSTCGLRQYSQPQFRIKGGLFADIASHPWQAAIFAKHRRSPGERFLCGGILISSCWILSAAHCFQERFPPHHLTVILGRTYRVVPGEEEQKFEVEKYIVHKEFDDDTYDNDIALLQLKSDSSRCAQESSVVRTVCLPPADLQLPDWTECELSGYGKHEALSPFYSERLKEAHVRLYPSSRCTSQHLLNRTVTDNMLCAGDTRSGGPQANLHDACQGDSGGPLVCLNDGRMTLVGIISWGLGCGQKDVPGVYTKVTNYLDWIRDNMRP	0.000023 mg/l	[64]
91	Proinsulin	*staphylococcal* protein A	MKKKNIYSIRKLGVGIASVTLGTLLISGGVTPAANAVDNKFNKEQQNAFYEILHLPNLNEEQRNAFIQSLKDDQSANLLAEAKKLNDAQAPKVDNKFNKEQQNAFYEILHLPNLNEEQRNAFIQSLKDDQSANLLAEAKKLNDAQAPKVDANSSSVPFVNQHLCGSHLVEALYLVCGERGFFYTPKTRREAEDLQVGQVELGGGPGAGSLQPLALEGSLQKRGIVEQCCTSICSLYQLENYCN	2.1 mg/l	[65]

92	Bovine pancreatic trypsin inhibitor (BPTI)	*staphylococcal* protein A	MKKKNIYSIRKLGVGIASVTLGTLLISGGVTPAANAAQHDEAQQNAFYQVLNMPNLNADQRNGFIQSLKDDPSQSANVLGEAQKLNDSQAPKADAQQNNFNKDQQSAFYEILNMPNLNEAQRNGFIQSLKDDPSQSTNVLGEAKKLNESQAPKADNNFNKEQQNAFYEILNMPNLNEEQRNGFIQSLKDDPSQSANLLSEAKKLNESQAPKADNKFNKEQQNAFYEILHLPNLNEEQRNGFIQSLKDDPSQSANLLAEAKKLNDAQAPKADNKFNKEQQNAFYEILHLPNLTEEQRNGFIQSLKDDPSVSKEILAEAKKLNDAQAPKRPDFCLEPPYTGPAKARIIRYFYNAKAGLCQTFVYGGARAKRNNFKSAEDCMRTCGGA	10 mg/l	[66]
93	human β_2 - microglobulin	ompA	MKKTAIAIAVALAGFATVAQAAEFLEAIQRTPKIQVYSRHPAENGKSNFLNCYVSGFHPSDIEVDLLKNGERIEKVEHSDLSFSKDWSFYLLYYTEFTPTEKDEYACRVNHVTLSNPKIVKWDRDM	10 mg/l	[67]
94	Interferon alpha2b	pelB	MKYLLPTAAAGLLLLAAQPAMAMCDLPQTHSLGSRRTLMLLAQMRRISLFSCLKDRHDFGFPQEEFGNQFQKAETIPVLHEMIQQIFNLFSTKDSSAAWDETLLDKFYTELYQQLNDLEACVIQGVGVTETPLMKEDSILAVRKYFQRITLYLKEKKYSPCAWEVVRAEIMRSFSLSTNLQESLRSKEGS*EQKLISEEDL*NS*HHHHHH*	< 1 mg/l	[42]
95	elicitin beta-cinnamomin from Phytophathora cinnamomi	MalE	MKIKTGARILALSALTTMMFSASALAHMTACTATQQTAAYKTLVSILSESSFSQCSKDSGYSMLTATALPTNAQYKLMCASTACNTMIKKIVALNPPDCDLTVPTSGLVLDVYTYANGFSSKCASLLEHHHHHH	17.6 mg/l	[68]
96	elicitin beta-cinnamomin from Phytophathora cinnamomi	PelB	MKYLLPTAAAGLLLLAAQPAMAHMTACTATQQTAAYKTLVSILSESSFSQCSKDSGYSMLTATALPTNAQYKLMCASTACNTMIKKIVALNPPDCDLTVPTSGLVLDVYTYANGFSSKCASLLEHHHHHH	13.3 mg/l	[68]

97	Human Growth Hormone (hGH)	PelB	MKYLLPTAAAGLLLLAAQPAMAMAAGSRTSLLLAFGLLCLSWLQEGSAFPTIPLSRLFDNAMLRARRLYQLAYDTYQEFEEAYILKEQKYSFLQNPQTSLCFSESIPTPSNRVKTQQKSNLELLRISLLLIQSWLEPVQLLRSVFANSLVYGASDSNVYRHLKDLEEGIQTLMWRLEDGSPRTGQIFNQSYSKFDTKSHNDDALLKNYGLLYCFRKDMDKVETFLRIVQCRSVEGSCGFLVPRGSLEHHHHHH	2.57 mg/lor 1.13 mg/l/OD_{600}	[69]
98	Aglycosylated recombinant human FcgammaRI	MalE	MKIKTGARILALSALTTMMFSASALA*KIEEAMG*QVDTTKAVITLQPPWVSVFQEETVTLHCEVLHLPGSSSTQWFLNGTATQTSTPSYRITSASVNDSGEYRCQRGLSGRSDPIQLEIHRGWLLLQVSSRVFTEGEPLALRCHAWKDKLVYNVLYYRNGKAFKFFHWNSNLTILKTNISHNGTYHCSGMGKHRYTSAGISVTVKELFPAPVLNASVTSPLLEGNLVTLSCETKLLLQRPGLQLYFSFYMGSKTLRGRNTSSEYQILTARREDSGLYWCEAATEDGNVLKRSPELELQVLGLQLPTPV*HHHHHH*	0.8 mg/l	[70]

Table S1: Sequence redundancy reduced dataset for training and testing of Periscope. ND – not detected

References

1 Le, Y., Peng, J., Wu, H., Sun, J. & Shao, W. An approach to the production of soluble protein from a fungal gene encoding an aggregation-prone xylanase in Escherichia coli. *PloS one* **6**, e18489 (2011).

2 Nausch, H. *et al.* Recombinant production of human interleukin 6 in Escherichia coli. *PloS one* **8**, e54933 (2013).

3 Larsen, M. W., Bornscheuer, U. T. & Hult, K. Expression of Candida antarctica lipase B in Pichia pastoris and various Escherichia coli systems. *Protein Expression and Purification* **62**, 90-97 (2008).

4 Soares, C. *et al.* Distinct human prolactin (hPRL) and growth hormone (hGH) behavior under bacteriophage lambda P L promoter control: Temperature plays a major role in protein yields. *Journal of Biotechnology* **133**, 27-35 (2008).

5 Förster, S. *et al.* Secretory expression of biologically active human Herpes virus interleukin-10 analogues in Escherichia coli via a modified Sec-dependent transporter construct. *BMC biotechnology* **13**, 82 (2013).

6 Kashimura, A. *et al.* Protein A-mouse acidic mammalian chitinase-V5-His expressed in periplasmic space of Escherichia coli possesses chitinase functions comparable to CHO-expressed protein. (2013).

7 Lobo, M. D. P. *et al.* Expression and efficient secretion of a functional chitinase from Chromobacterium violaceum in Escherichia coli. *BMC biotechnology* **13**, 46 (2013).

8 Caldinelli, L., Albani, D. & Pollegioni, L. One single method to produce native and Tat-fused recombinant human α-synuclein in Escherichia coli. *BMC biotechnology* **13**, 32 (2013).

9 Heel, T., Paal, M., Schneider, R. & Auer, B. Dissection of an old protein reveals a novel application: domain D of Staphylococcus aureus Protein A(sSpAD) as a secretion- tag. *Microbial cell factories* **9**, 92-92 (2010).

10 Hewinson, R., Harris, D., Whelan, A. & Russell, W. Secretion of the mycobacterial 19-kilodalton protein by *Escherichia coli*, a novel method for the purification of recombinant mycobacterial antigens. *Clinical and diagnostic laboratory immunology* **3**, 23-29 (1996).
11 Dresler, K., van den Heuvel, J., Müller, R.-J. & Deckwer, W.-D. Production of a recombinant polyester-cleaving hydrolase from *Thermobifida fusca* in *Escherichia coli*. *Bioprocess and Biosystems Engineering* **29**, 169-183 (2006).
12 Fisher, A. C. *et al.* Exploration of twin-arginine translocation for expression and purification of correctly folded proteins in *Escherichia coli*. *Microbial biotechnology* **1**, 403-415 (2008).
13 Aguilar-Yáñez, J. M. *et al.* An influenza A/H1N1/2009 hemagglutinin vaccine produced in *Escherichia coli*. *PloS one* **5**, e11694 (2010).
14 Anangi, R., Rash, L. D., Mobli, M. & King, G. F. Functional expression in *Escherichia coli* of the disulfide-rich sea anemone peptide APETx2, a potent blocker of acid-sensing ion channel 3. *Marine drugs* **10**, 1605-1618 (2012).
15 Cossins, A. J., Harrison, S., Popplewell, A. G. & Gore, M. G. Recombinant production of a V L single domain antibody in *Escherichia coli* and analysis of its interaction with peptostreptococcal protein L. *Protein Expression and Purification* **51**, 253-259 (2007).
16 Singh, P. *et al.* Effect of signal peptide on stability and folding of *Escherichia coli* thioredoxin. (2013).
17 Songsiriritthigul, C., Buranabanyat, B., Haltrich, D. & Yamabhai, M. Efficient recombinant expression and secretion of a thermostable GH26 mannan endo-1, 4-β-mannosidase from *Bacillus licheniformis* in *Escherichia coli*. *Microbial cell factories* **9**, 20 (2010).
18 Cai, M., Zhu, F. & Shen, P. Expression and purification of chicken beta interferon and its antivirus immunological activity. *Protein Expression and Purification* **84**, 123-129 (2012).
19 Sinsuwan, S., Yongsawatdigul, J., Chumseng, S. & Yamabhai, M. Efficient expression and purification of recombinant glutaminase from *Bacillus licheniformis* (GlsA) in *Escherichia coli*. *Protein Expression and Purification* **83**, 52-58 (2012).
20 Sarduy, E. S., Muñoz, A. C., Trejo, S. A. & Planes, M. d. l. A. C. High-level expression of Falcipain-2 in *Escherichia coli* by codon optimization and auto-induction. *Protein Expression and Purification* **83**, 59-69 (2012).
21 Maîtrepierre, E., Sigoillot, M., Le Pessot, L. & Briand, L. Recombinant expression, in vitro refolding, and biophysical characterization of the N-terminal domain of T1R3 taste receptor. *Protein Expression and Purification* **83**, 75-83 (2012).
22 Qian, L. *et al.* Expression and purification of recombinant human Mig in *Escherichia coli* and its comparison with murine Mig. *Protein Expression and Purification* **82**, 205-211 (2012).
23 Takemori, D., Yoshino, K., Eba, C., Nakano, H. & Iwasaki, Y. Extracellular production of phospholipase A 2 from *Streptomyces violaceoruber* by recombinant *Escherichia coli*. *Protein Expression and Purification* **81**, 145-150 (2012).
24 Zhou, W. *et al.* Prokaryotic expression and bioactivity analysis of N-terminus domain of *Pinellia ternata* agglutinin using alkaline phosphatase signal peptide. *Protein Expression and Purification* **89**, 84-91 (2013).
25 Zelena, K., Krings, U. & Berger, R. G. Functional expression of a valencene dioxygenase from *Pleurotus sapidus* in *E. coli*. *Bioresource technology* **108**, 231-239 (2012).
26 Tiwari, A., Sankhyan, A., Khanna, N. & Sinha, S. Enhanced periplasmic expression of high affinity humanized scFv against Hepatitis B surface antigen by codon optimization. *Protein Expression and Purification* **74**, 272-279 (2010).

27 Stampolidis, P., Kaderbhai, N. N. & Kaderbhai, M. A. Periplasmically-exported lupanine hydroxylase undergoes transition from soluble to functional inclusion bodies in *Escherichia coli*. *Archives of biochemistry and biophysics* **484**, 8-15 (2009).
28 Tu, W. *et al.* Improved production of holotoxin Stx2 with biological activities by using a single-promoter vector and an auto-induction expression system. *Protein Expression and Purification* **67**, 169-174 (2009).
29 Tudyka, T. & Skerra, A. Glutathione S-transferase can be used as a C-terminal, enzymatically active dimerization module for a recombinant protease inhibitor, and functionally secreted into the periplasm of *Escherichia coli*. *Protein Science* **6**, 2180-2187 (1997).
30 French, C., Keshavarz-Moore, E. & Ward, J. M. Development of a simple method for the recovery of recombinant proteins from the *Escherichia coli* periplasm. *Enzyme and Microbial Technology* **19**, 332-338 (1996).
31 Johansson, H., Jägersten, C. & Shiloach, J. Large scale recovery and purification of periplasmic recombinant protein from *E. coli* using expanded bed adsorption chromatography followed by new ion exchange media. *Journal of Biotechnology* **48**, 9-14 (1996).
32 Bishai, W. R., Rappuoli, R. & Murphy, J. R. High-level expression of a proteolytically sensitive diphtheria toxin fragment in *Escherichia coli*. *Journal of bacteriology* **169**, 5140-5151 (1987).
33 Oka, T. *et al.* Synthesis and secretion of human epidermal growth factor by *Escherichia coli*. *Proceedings of the National Academy of Sciences* **82**, 7212-7216 (1985).
34 Chen, Y.-C., Chen, L.-A., Chen, S.-J., Chang, M.-C. & Chen, T.-L. A modified osmotic shock for periplasmic release of a recombinant creatinase from *Escherichia coli*. *Biochemical engineering journal* **19**, 211-215 (2004).
35 Vuori, K., Myllylä, R., Pihlajaniemi, T. & Kivirikko, K. I. Expression and site-directed mutagenesis of human protein disulfide isomerase in Escherichia coli. This multifunctional polypeptide has two independently acting catalytic sites for the isomerase activity. *Journal of Biological Chemistry* **267**, 7211-7214 (1992).
36 Hussack, G. *et al.* Neutralization of *Clostridium difficile* toxin A with single-domain antibodies targeting the cell receptor binding domain. *Journal of Biological Chemistry* **286**, 8961-8976 (2011).
37 Barth, S. *et al.* Compatible-solute-supported periplasmic expression of functional recombinant proteins under stress conditions. *Applied and Environmental Microbiology* **66**, 1572-1579 (2000).
38 Barrett, C. M. L., Ray, N., Thomas, J. D., Robinson, C. & Bolhuis, A. Quantitative export of a reporter protein, GFP, by the twin-arginine translocation pathway in Escherichia coli. *Biochemical and Biophysical Research Communications* **304**, 279-284 (2003).
39 Uchida, H. *et al.* Secretion of authentic 20-kDa human growth hormone (20K hGH) in Escherichia coli and properties of the purified product. *Journal of Biotechnology* **55**, 101-112 (1997).
40 Medina-Rivero, E. *et al.* Modified penicillin acylase signal peptide allows the periplasmic production of soluble human interferon-γ but not of soluble human interleukin-2 by the Tat pathway in Escherichia coli. *Biotechnology Letters* **29**, 1369-1374, doi:10.1007/s10529-007-9395-5 (2007).
41 Becker, G. W. & Hsiung, H. M. Expression, secretion and folding of human growth hormone in Escherichia coli. Purification and characterization. *FEBS Letters* **204**, 145-150 (1986).

42 Sletta, H. *et al.* The presence of N-terminal secretion signal sequences leads to strong stimulation of the total expression levels of three tested medically important proteins during high-cell-density cultivations of Escherichia coli. *Applied and Environmental Microbiology* **73**, 906-912, doi:10.1128/aem.01804-06 (2007).
43 Hernandez, V. E. B. *et al.* Periplasmic expression and recovery of human interferon gamma in Escherichia coli. *Protein Expression and Purification* **59**, 169-174, doi:10.1016/j.pep.2008.01.019 (2008).
44 Chatel, J. M., Adel-Patient, K., Créminon, C. & Wal, J. M. Expression of a lipocalin in prokaryote and eukaryote cells: Quantification and structural characterization of recombinant bovine β-lactoglobulin. *Protein Expression and Purification* **16**, 70-75 (1999).
45 Hasenwinkle, D. *et al.* Very high-level production and export in Escherichia coli of a cellulose binding domain for use in a generic secretion-affinity fusion system. *Biotechnology and Bioengineering* **55**, 854-863 (1997).
46 Hammarberg, B. *et al.* Dual affinity fusion approach and its use to express recombinant human insulin-like growth factor II. *Proceedings of the National Academy of Sciences of the United States of America* **86**, 4367-4371 (1989).
47 Humphreys, D. P. *et al.* High-level periplasmic expression in Escherichia coli using a eukaryotic signal peptide: Importance of codon usage at the 5 ' end of the coding sequence. *Protein Expression and Purification* **20**, 252-264, doi:10.1006/prep.2000.1286 (2000).
48 Kipriyanov, S. M., Moldenhauer, G. & Little, M. High level production of soluble single chain antibodies in small-scale Escherichia coli cultures. *Journal of Immunological Methods* **200**, 69-77, doi:10.1016/s0022-1759(96)00188-3 (1997).
49 Greenberg, R. *et al.* Expression of biologically active, mature human granulocyte-macrophage colony stimulating factor with an E. coli secretory expression system. *Current Microbiology* **17**, 321-332 (1988).
50 Lee, S. J., Kim, I. C., Kim, D. M., Bae, K. H. & Byun, S. M. High level secretion of recombinant staphylokinase into periplasm of Escherichia coli. *Biotechnology Letters* **20**, 113-116, doi:10.1023/a:1005359920522 (1998).
51 Kaderbhai, M. A., Ugochukwu, C. C., Kelly, S. L. & Lamb, D. C. Export of Cytochrome P450 105D1 to the Periplasmic Space of Escherichia coli. *Applied and Environmental Microbiology* **67**, 2136-2138 (2001).
52 Jeong, K. J. & Lee, S. Y. Secretory production of human granulocyte colony-stimulating factor in Escherichia coli. *Protein Expression and Purification* **23**, 311-318 (2001).
53 Jeong, K. J. & Lee, S. Y. Secretory production of human leptin in Escherichia coli. *Biotechnology and Bioengineering* **67**, 398-407 (2000).
54 Guisez, Y. *et al.* Efficient secretion of biologically active recombinant OB protein (leptin) in Escherichia coli, purification from the periplasm and characterization. *Protein Expression and Purification* **12**, 249-258 (1998).
55 Chung, B. H. *et al.* Overproduction of human granulocyte-colony stimulating factor fused to the PelB signal peptide in Escherichia coli. *Journal of Fermentation and Bioengineering* **85**, 443-446 (1998).
56 Choi, J. H., Jeong, K. J., Kim, S. C. & Lee, S. Y. Efficient secretory production of alkaline phosphatase by high cell density culture of recombinant Escherichia coli using the Bacillus sp. endoxylanase signal sequence. *Applied Microbiology and Biotechnology* **53**, 640-645 (2000).
57 de Taxis du Poet, P. *et al.* Production of the HV1 variant of hirudin by recombinant DNA methodology. *Blood coagulation & fibrinolysis : an international journal in haemostasis and thrombosis* **2**, 113-120 (1991).

58 Carter, P. *et al.* High level Escherichia coli expression and production of a bivalent humanized antibody fragment. *Nature Biotechnology* **10**, 163-167 (1992).
59 Kim, J. Y. *et al.* Twin-arginine translocation of active human tissue plasminogen activator in Escherichia coli. *Applied and Environmental Microbiology* **71**, 8451-8459 (2005).
60 Kaderbhai, M. A., Ugochukwu, C. C., Lamb, D. C. & Kelly, S. L. Targeting of active human cytochrome P4501A1 (CYP1A1) to the periplasmic space of Escherichia coli. *Biochemical and Biophysical Research Communications* **279**, 803-807 (2000).
61 Jobling, M. G., Palmer, L. M., Erbe, J. L. & Holmes, R. K. Construction and characterization of versatile cloning vectors for efficient delivery of native foreign proteins to the periplasm of Escherichia coli. *Plasmid* **38**, 158-173 (1997).
62 Loo, T., Patchett, M. L., Norris, G. E. & Lott, J. S. Using secretion to solve a solubility problem: High-yield expression in Escherichia coli and purification of the bacterial glycoamidase PNGase F. *Protein Expression and Purification* **24**, 90-98 (2002).
63 Winter, J., Neubauer, P., Glockshuber, R. & Rudolph, R. Increased production of human proinsulin in the periplasmic space of Escherichia coli by fusion to DsbA. *Journal of Biotechnology* **84**, 175-185 (2000).
64 Schäffner, J., Winter, J., Rudolph, R. & Schwarz, E. Cosecretion of Chaperones and Low-Molecular-Size Medium Additives Increases the Yield of Recombinant Disulfide-Bridged Proteins. *Applied and Environmental Microbiology* **67**, 3994-4000 (2001).
65 Yoon, J. W. Effect of modification of connecting peptide of proinsulin on its export. *Journal of Biotechnology* **36**, 45-54 (1994).
66 Nilsson, B. & Abrahmsén, L. [13] Fusions to staphylococcal protein A. *Methods in Enzymology* **185**, 144-161, doi:http://dx.doi.org/10.1016/0076-6879(90)85015-G (1990).
67 Parker, K. C. & Wiley, D. C. Overexpression of native human β-microglobulin in Escherichia coli and its purification. *Gene* **83**, 117-124, doi:http://dx.doi.org/10.1016/0378-1119(89)90409-5 (1989).
68 Hofzumahaus, S. & Schallmey, A. Escherichia coli-based expression system for the heterologous expression and purification of the elicitin β-cinnamomin from Phytophthora cinnamomi. *Protein Expression and Purification* **90**, 117-123, doi:http://dx.doi.org/10.1016/j.pep.2013.05.010 (2013).
69 Sockolosky, J. T. & Szoka, F. C. Periplasmic production via the pET expression system of soluble, bioactive human growth hormone. *Protein Expression and Purification* **87**, 129-135, doi:http://dx.doi.org/10.1016/j.pep.2012.11.002 (2013).
70 Hatayama, K., Asaoka, Y., Hoya, M. & Ide, T. Effective expression of soluble aglycosylated recombinant human Fcγ receptor i by low translational efficiency in Escherichia coli. *Applied Microbiology and Biotechnology* **94**, 1051-1059 (2012).
